# Examining the effect of expected test format and test difficulty on the frequency and mnemonic costs of mind wandering

**DOI:** 10.1177/17470218231187892

**Published:** 2023-07-21

**Authors:** Skylar J Laursen, Jeffrey D Wammes, Chris M Fiacconi

**Affiliations:** 1 Department of Psychology, University of Guelph, Guelph, Ontario, Canada; 2 Department of Psychology, Queen’s University, Kingston, Ontario, Canada; 3 Centre for Neuroscience Studies, Queen’s University, Kingston, Ontario, Canada

**Keywords:** Mind wandering, test format, test difficulty, memory

## Abstract

Mind wandering, generally defined as task-unrelated thought, has been shown to constitute between 30% and 50% of individuals’ thoughts during almost every activity in which they are engaged. Critically, however, previous research has shown that the demands of a given task can lead to either the up- or down-regulation of mind wandering and that engagement in mind wandering may be differentially detrimental to future memory performance depending on learning conditions. The goal of the current research was to gain a better understanding of how the circumstances surrounding a learning episode affect the frequency with which individuals engage in off-task thought, and the extent to which these differences differentially affect memory performance across different test formats. Specifically, while prior work has manipulated the conditions of encoding, we focused on the anticipated characteristics of the retrieval task, thereby examining whether the anticipation of later demands imposed by the expected test format/difficulty would influence the frequency or performance costs of mind wandering during encoding. Across three experiments, we demonstrate that the anticipation of future test demands, as modelled by expected test format/difficulty, does not affect rates of mind wandering. However, the costs associated with mind wandering do appear to scale with the difficulty of the test. These findings provide important new insights into the impact of off-task thought on future memory performance and constrain our understanding of the strategic regulation of inattention in the context of learning and memory.

When completing a task or activity, it is very common for one to find themselves drifting towards unrelated thoughts, such as something they did previously or plan to do later, or their personal worries or daydreams. This commonly reported form of off-task thinking has generally been referred to as mind wandering (Smallwood & Schooler, 2006) and constitutes between 30% and 50% of individuals’ thoughts during almost every activity in which they are engaged (Killingsworth & Gilbert, 2012). A large body of research has now demonstrated that there are significant costs associated with off-task thought across multiple domains, including decreased reading comprehension (Franklin et al., 2011; Pachai et al., 2016; Riechle et al., 2010), impoverished lecture learning (Kane et al., 2017; Risko et al., 2012; Wammes, Seli, et al., 2016; Wammes & Smilek, 2017), lower working memory capacity (Mrazek et al., 2012), and memory impairments (Blondé et al., 2022; Maillet & Rajah, 2013; Smallwood, Baracaia, et al., 2003; Thomson, Smilek, & Besner, 2014), as well as increased risk of driving mistakes and accidents (He et al., 2011; Yanko & Spalek, 2013, 2014), increases in response variability (Anderson et al., 2021; Laflamme et al., 2018; Seli et al., 2013), commission errors on the Sustained Attention to Response Task (SART; Christoff et al., 2009), slower sequence learning (Franklin et al., 2015), and reduced happiness (Killingsworth & Gilbert, 2012; see Mooneyham & Schooler, 2013 for a review). Taken together, these findings clearly display that mind wandering, and inattention can have detrimental effects across a variety of tasks. However, it is also likely that the rate of mind wandering will vary dramatically across these tasks, and perhaps more importantly, so too will the manner in which performance deficits present. Therefore, it is important to probe whether rates of mind wandering and the associated costs differ depending on the characteristics of the task being completed. Here, we use the domain of learning and memory as a testing ground, as it is well known that different episodic memory tests vary in their processing demands, which may translate into potential differences in the way in which individuals prepare for such tests. Our goal is to gain a better understanding of how the circumstances surrounding a learning episode affect the frequency with which individuals engage in off-task thought, and the extent to which off-task thought differentially affects memory performance across different memory test formats.

A few established factors are known to influence the incidence of mind wandering generally. For instance, rates of mind wandering tend to increase as the amount of time spent completing a task increases (Farley et al., 2013; Kane et al., 2017, 2021; Metcalfe & Xu, 2016; Risko et al., 2012; Thomson, Seli et al., 2014; Wammes & Smilek, 2017), as well as when interest in and motivation to complete a task decreases (Kane et al., 2017; Lindquist & McLean, 2011; Seli et al., 2017; Seli, Wammes, et al., 2015; Unsworth & McMillan, 2013; Varao-Sousa & Kingstone, 2015). Importantly, previous research has also demonstrated that the task context also plays a role in attention fluctuations over time—rates of mind wandering increased over time during a lecture in previous work, but only when it was presented in video format (i.e., not live; Wammes & Smilek, 2017). In another example, participants strategically reduced their mind wandering in anticipation of an upcoming target (Seli, Carriere, et al., 2018). More specific to the purposes of the current work, differences in task demands during memory encoding could change mind wandering rates and their consequences. This dependency has been established by work showing that massed studying during encoding (relative to spaced studying) results in higher mind wandering and reduced memory performance (Metcalfe & Xu, 2016). Similarly, other work found that mind wandering was negatively correlated with recognition memory performance when participants completed a deep, but not shallow, processing task during encoding (Thomson, Smilek, & Besner, 2014).

Critically, these findings highlight the idea that the specific demands of a learning event can lead to either the up- or down-regulation of mind wandering and that engagement in mind wandering may be differentially detrimental to future memory performance depending on learning conditions (Maillet et al., 2017; Metcalfe & Xu, 2016; Seli, Carriere, et al., 2018; Seli, Konishi, et al., 2018; Thomson, Smilek, & Besner, 2014; Wammes & Smilek, 2017). Following this, there is reason to suspect that other conditions of learning, namely, expected test format/difficulty, may exert similar effects on the rate and costs of mind wandering. Although prior work has manipulated the conditions of encoding (e.g., spacing: Metcalfe & Xu, 2016; depth: Thomson, Smilek, & Besner, 2014), we focused on the anticipated characteristics of the retrieval task. Specifically, we were interested in whether the anticipation of later demands imposed by expected test format/difficulty would influence the frequency or performance costs of mind wandering during encoding.

Indeed, previous research has shown that after gaining experience with different test formats, individuals begin to adjust their encoding strategies to match the specific demands of a given test (Cho & Neely, 2017; Finley & Benjamin, 2012; Rivers & Dunlosky, 2020). For example, some groups used test-expectancy paradigms in which participants completed many study-test cycles of either cued- or free-recall tests. This led participants to develop an expectation for the type of test that they had been completing. During the final study-test cycle, however, participants received a test that either matched (e.g., expecting cued-recall and receive cued-recall) or mismatched (e.g., expecting cued-recall and received free-recall) their expectations (Finley & Benjamin, 2012, Experiment 2; Rivers & Dunlosky, 2020, Experiment 1). Critically, memory performance on the final test was greater when the type of test matched the participants’ expectations. The authors attributed this performance advantage to the encoding strategies used by participants (Finley & Benjamin, 2012, Experiment 2; Rivers & Dunlosky, 2020, Experiment 1). Specifically, when participants were led to expect a cued-recall test they reported relying on cue-target associations as an encoding strategy. In contrast, when they were led to expect a free-recall test, participants instead reported using target-target associations, target focus, and rote rehearsal (Finley & Benjamin, 2012, Experiment 2; Rivers & Dunlosky, 2020, Experiment 1).

Based on the literature reviewed thus far, two conclusions can be drawn. First, we know that rates of mind wandering and potentially the costs of mind wandering vary due to task demands (Seli, Carriere, et al., 2018). Second, we know that learners can tailor their encoding strategies to match the demands of an upcoming memory test (Cho & Neely, 2017; Finley & Benjamin, 2012; Rivers & Dunlosky, 2020). Together, these findings raise the possibility that one avenue through which individuals may shift their encoding is by strategically allocating greater attention during encoding based on the anticipated test demands. Individuals may differentially engage in mind wandering analogous to how they engage in differential encoding and study strategies as a function of the expected test format. When preparing for a test that represents previously seen information (e.g., forced-choice recognition), a relatively easy test of memory, individuals may be more inclined to mind wandering, as compared with when preparing for a test that is generative in nature (e.g., cued-recall), a relatively difficult test of memory. This reasoning is supported by a recent study from Middlebrooks et al. (2017). Here, they built expectations for either a free-recall, or a recognition test, over four study-test blocks. In these blocks, studied words were assigned point values (between 1 and 10), and participants were to remember as many words, and therefore earn as many points, as possible. They then gave both groups a fifth study-test block that involved free-recall. The group that was anticipating a recognition test recalled significantly less high-value items than those anticipating recall, despite having overall high recognition performance in previous test phases. The authors suggested that when anticipating a recognition test, individuals may be less attentive to item details (e.g., point-value), seeing as the ease of the test allows for high overall performance (Middlebrooks et al., 2017). This strategy, designed to support recognition, is counterproductive when preparing for free-recall tests because the difficulty of the test requires strategic engagement with high-value items to maximise memory for these items. As such, it may be that the resources devoted to items at encoding is driven by the difficulty and demands of the anticipated test format. These findings suggest that individuals may engage in mind wandering more often when preparing for a relatively easy test format (e.g., forced-choice recognition) but that doing so may not be as detrimental to memory performance as when preparing for a relatively difficult test format requiring greater attentional resources during encoding (e.g., cued-recall). In other words, mind wandering at encoding may yield less of a cost on a subsequent recognition memory test because the increased retrieval support may offset any deficits in encoding accrued through mind wandering.

Indeed, some theoretical accounts of mind wandering, such as the resource-control account (Thomson et al., 2015), have proposed that the attentional resources devoted to mind wandering and task performance are in direct competition (Smallwood, 2010; Smallwood & Schooler, 2006; Thomson, Smilek, & Besner, 2014). When engaging in mind wandering, a common attentional resource is divided between internal and external information (Smallwood, 2010; Smallwood, Obonsawin, et al., 2003). Thus, when an individual engages in mind wandering, rather than focusing on external sensory information, attention instead shifts towards internal thoughts and feelings (Smallwood, 2010). Related forms of divided attention have been shown to be detrimental when attempting to encode important information (e.g., Baddeley et al., 1984; Craik et al., 1996), as having fewer available resources reduces the formation of detailed episodic memories that support recollection (Jennings & Jacoby, 1993; see Yonelinas, 2002, for a review). Critically however, it has also been shown that when attention is divided during encoding, as is the case when engaging in off-task thought, retrieval of information can occur on the basis of familiarity rather than recollection (Gardiner et al., 2001; Gardiner & Parkin, 1990; Jacoby et al., 1989; Smallwood, Baracaia, et al., 2003). Given that some tests of memory can be completed on the basis of familiarity (e.g., item recognition), while others rely more heavily on the ability to recollect specific information (e.g., free-recall), it is not surprising that divided attention during encoding has been shown to produce greater costs when the latter type of test is completed (Craik et al., 1996). The question remains, however, are the mnemonic costs associated with mind wandering analogous to those of divided attention? That is, are there greater performance costs associated with mind wandering when the expected test format relies on individuals’ ability to recollect specific information (i.e., cued-recall) rather than simply recognise material they are familiar with (i.e., forced-choice recognition)?

One final point to consider is the way in which individuals engage in mind wandering. Specifically, researchers have often distinguished between intentional and unintentional mind wandering, where the former refers to deliberate engagement in off-task thought by an individual, and the latter refers to spontaneous occurrences of off-task thought outside an individual’s control (Isacescu et al., 2017; Martarelli et al., 2021; Seli, Carriere, et al., 2015; Seli et al., 2016). Importantly, because these two forms of mind wandering are associated with different processes, the frequency with which individuals engage in these two forms of mind wandering may be impacted by the expected test format differently. Specifically, given that intentional mind wandering is under conscious control (Seli, Carriere, et al., 2015; Seli et al., 2016), an individual may be more likely to tailor how often they intentionally engage in off-task thought based on expected test format. However, because individuals do not choose when they engage in unintentional mind wandering, there may be little, if any, impact of expected test format/difficulty on the frequency of individual’s reports of unintentional mind wandering. In addition to the frequency of off-task thought, the mnemonic impact of off-task thought may also depend on whether such off-task thought was intentional or unintentional. Given that individuals do not regulate when they engage in unintentional mind wandering, such mind wandering may almost always yield a subsequent mnemonic cost that will be proportional to the demands of a specific testing method (i.e., cued-recall vs. recognition). However, because individuals have the ability to regulate when they engage in intentional off-task thought, they may choose to strategically mind wandering at times when it is less likely to affect encoding. As a consequence, memory performance may be less impacted overall, and some forms of memory that rely on gist-based information could be left fully intact (e.g., item recognition). However, this possibility assumes that individuals can accurately gauge when such intentional mind wandering would be the least costly. To the extent that they are not able to accurately make this assessment, the mnemonic cost of intentional mind wandering may be similar to that of unintentional mind wandering and be subject to the retrieval demands of a specific testing method.

## Overview of current research

From the literature reviewed thus far, there is clear evidence that engagement in off-task thought can be modulated and is influenced by many factors, some of which are specific to memory encoding. These include lecture format, time-on-task, massed vs spaced learning, task context and demand, and task difficulty (Metcalfe & Xu, 2016; Risko et al., 2012; Seli, Carriere, et al., 2018; Seli, Konishi, et al., 2018; Thomson et al., 2015; Thomson, Seli, et al., 2014; Wammes & Smilek, 2017). However, it is yet unknown whether the anticipated demands of a retrieval test affect the frequency with which individuals engage in off-task thought. Moreover, it remains unclear whether engaging in off-task thought affects memory performance differentially for different test formats. These questions are important given the need for appropriate and effective learning strategies when preparing for future memory tasks. In addition, given the emphasis placed on performance in academic settings, understanding how and when individuals attend to stimuli may provide new insights that help to promote greater task engagement. The purpose of the current study is twofold: first, we sought to examine whether expected test format influences the frequency with which individuals engage in off-task thought during the study (Experiment 1), and second, we sought to investigate whether engagement in off-task thought is differentially detrimental to memory performance for differing test formats and difficulties (Experiments 1, 2, and 3). To foreshadow, we found that anticipation of future test demands, as modelled by expected test format/difficulty, does not affect rates of mind wandering. However, the costs associated with mind wandering do appear to scale with the difficulty of the test.

## Experiment 1

Here, we report our initial attempt at investigating whether individuals strategically regulate their off-task thoughts when preparing for different test formats and whether this off-task thought differentially affects memory performance. In our design, participants completed six study-test cycles, which alternated between forced-choice recognition (i.e., a test that relies heavily on familiarity) and cued-recall tests (i.e., a test that relies on generative memory). During each study phase, we measured rates of intentional and unintentional mind wandering using thought probes, then compared the frequency of mind wandering between the test formats. We also explored whether the mnemonic costs of mind wandering differed between forced-choice recognition and cued-recall tests. Insofar as individuals purposefully engage in off-task thought during easy tasks (e.g., encoding memories that rely on familiarity), mind wandering should be more frequent in forced-choice recognition relative to cued-recall tests. Furthermore, to the extent that mind wandering involves reductions in attention and resource allocation like those involved with divided attention (Craik et al., 1996), mind wandering during encoding should impair cued-recall memory performance more than forced-choice recognition.

### Method

All methods and statistical analyses were preregistered (aspredicted.org; reference #51027). Any deviations from the preregistered analysis plan (i.e., excluded or additional analyses) are noted and justified in the text.

#### Participants

Participants (*N* = 249) were recruited from the University of Guelph psychology participant pool (*n* = 155; *Mean* age = 18.52, *SD* = 1.32, 120 female) and Prolific (2020; *n* = 94; *Mean* age = 23.66, *SD* = 4.05, 33 female, 39 identifying as students). Participants from the University of Guelph psychology participant pool received partial course credit and those from Prolific received monetary compensation ($15 CND) in exchange for their participation.^1^ Participants were eligible for the study if they had English proficiency, were between the ages of 18 and 35 years of age, and had normal or corrected to normal vision. No geographic exclusions were used in Prolific participant recruitment (79% from Europe, 14% from North America, 3% from Asia, 1% from South America and Africa, and 2% unknown).

Prior to analyses, exclusions were applied based on participants’ answers to questions used to probe participants’ understanding of the instructions given and their general engagement with the task (see the online Supplementary Material A and Experiment 1 Procedure for more detail). All exclusion criteria were preregistered, and exclusions were performed blind to whether data conformed to experimental predictions. Twenty-four participants (five from Prolific) who incorrectly responded to five or more (~40% or more, see Laursen & Fiacconi, 2022) out of the 14 basic questions about the instructions on the first try were excluded. One hundred and twenty-five participants (61 from Prolific) who indicated they wrote down word pairs during the study phase, were using their cellphone throughout the study, or indicated they left the room at some point during the study were also excluded. This left a total of 100 participants (42 from Prolific) in the final sample, as intended in our preregistration. The overall exclusion rate was 60% (54% from the University sample, 70% from Prolific).

#### Materials

This experiment was programmed using PsychoPy software (Peirce et al., 2019) and was made accessible to participants through Pavlovia (2020). Participants completed the experiment online using their own personal computers and were unable to access the experiment on their cellphone or tablet.

Stimuli consisted of 270 weakly related cue-target word pairs (e.g., *tent-forest, button-pocket;* see the online Supplementary Material—Table B1 for the full set). Using a similar procedure as Laursen and Fiacconi (2021), a list of words was generated using the MRC Psycholinguistic Database with the following parameters: Number of letters: 3–8; Kucera–Francis Written Frequency: 10–300; Concreteness Rating: 300–700; Imageability Rating: 300–700. Next, following prior work (Laursen & Fiacconi, 2021), the experimenters manually generated the word pairs such that the two words could be related using a *connecting word*. For example, the words *sky* and *ocean* can be linked through the connecting word, *blue*, because both the sky and the ocean are blue. The latent semantic analysis (LSA) value for each word pair was calculated using the LSAfun package for *R* (Günther et al., 2015). LSA values reflect how often the two words appear together in the English language and were used to quantify the degree to which members of a word pair were related.

Participants were presented with six lists, each consisting of 45 pairs of words, across six alternating cued-recall and forced-choice recognition study-test cycles. An independent samples analysis of variance (ANOVA) was conducted to ensure the mean LSA values for word pairs did not differ across lists (see Supplementary Material Table B2 for statistical output). Ordering of the lists presented was randomised, as was the test format completed in the first study-test cycle (i.e., cued-recall or forced-choice recognition). Furthermore, the order of the word pairs was randomised within each list.

#### Procedure

Prior to beginning the experiment, participants were asked to turn off their cellphone, email, and music to minimise distractions. After providing informed consent, participants were given general instructions regarding what was required of them during the experiment as well as a brief definition of mind wandering (see Supplementary Material C for a detailed description of the instructions given to participants regarding mind wandering).

##### Study phase

In study phases preceding cued-recall tests, participants were told they would be preparing for a cued-recall test and that this would require them to correctly recall from memory the target (right) word when later presented with the cue (left) word. In study phases preceding forced-choice recognition tests, participants were told they would be preparing for a forced-choice recognition test and that this would require them to correctly select the target (right) word that was previously paired with the cue (left) word from a set of three different options. Instructions for each study phase were otherwise identical, except that after the instructions for the first study cycle, participants had to answer four comprehension questions about them (see Supplementary Material A). All other study phases only required participants to correctly respond to the question of which test format they would be preparing for (i.e., cued-recall or forced-choice recognition). Participants could not begin the study phase until each question had been answered correctly. Each trial began with a 1000 ms central fixation cross, followed by the presentation of the to-be-learned word pair in the centre of the screen for 5 s.

We periodically assessed mind wandering by pseudo-randomly placing thought probes throughout the study phases. On average, these probes occurred once every 45 s, though the possible durations between probes could be 35, 40, 45, 50 or 55 s, with the exact placement chosen randomly. When a thought probe occurred, participants were asked to select the response that best characterised their mental state just before the thought probe was presented. Participants could select one of three options: (1) on-task, (2) off-task: intentional, or (3) off-task: unintentional. Participants’ responses to thought probes were self-paced and the study phase continued immediately following their selection. Because the probe presentation was completely random, it was possible for thought probes to appear mid-trial (i.e., before the pair had been on screen for the allotted 5 s). For these trials, the word pair was presented for the remainder of the time before the next word pair was presented. For example, if a probe appeared 2.3 s into a pair presentation, the pair was shown for another 2.7 s after the participant responded to the probe.

##### Test phase

Before each test phase, participants completed a 1 min math (distractor) task, which was identical across all study-test cycles. Following the distractor task, participants then completed a self-paced cued-recall or forced-choice recognition test, depending on the cycle. Like the study phase, participants were required to answer instruction comprehension questions prior to the first test phase (see Supplementary Material A) and could not advance without answering them correctly. Other than these clarification questions, instructions within each test format were identical.

##### Cued-recall

Each trial began with a 500 ms central fixation cross followed by the presentation of one of the cue words from the previous study phase on the left side of the screen and an empty textbox on the right. Participants were asked to recall the corresponding target word as accurately as possible and type it into the textbox using their keyboard. Each trial was self-paced, and participants could advance to the next trial by pressing the Enter key.

##### Forced-choice recognition

Each trial began with a 500 ms central fixation cross followed by the presentation of one of the cue words from the previous study phase on the left side of the screen. Three words appeared on the right side of the screen in a columnar fashion, the corresponding target word and two unrelated lure words. The two lure items were novel words not previously encountered in the prior study phase and were unrelated to the cue and target words. Novel, unrelated lures were chosen to maximise the likelihood that participants would perceive the forced-choice recognition test to be easier. The position of lure and target words (top, centre, bottom) was randomised on each trial. As well, pairing of the two lure words and correct target words was randomised for each participant. Participants were required to select the correct target word using the 1, 2, or 3, keys on their keyboard which corresponded to the top, middle, and bottom positions, respectively. Each trial was self-paced, and the next trial began immediately after one of the three allowed keys were pressed.

After completion of either the cued-recall or forced-choice recognition test, participants had completed one study-test cycle. Participants then completed five additional study-test cycles alternating between cued-recall and forced-choice recognition tests. After completion of the final study-test cycle, participants were asked two follow-up questions as to whether they wrote anything down during either study phase to help with memory performance, or if they experienced any interruptions during the experiment (e.g., cellphone, another person in the room, leaving the experiment at any time; see Supplementary Material A). If participants reported any of these interruptions, they were asked whether they had reported these as instances of being off-task when asked. Following these questions, participants were debriefed and thanked for their participation.

### Results

All analyses were conducted using R software (R Core Team, 2017), and a significance level of .05 was adopted. Effect sizes reported were calculated using test statistics and odds ratio conversions using the *effectsize* package for *R* (Ben-Shachar et al., 2020), and any violations of sphericity were corrected using the Huynh–Feldt adjustment. We begin by reporting the frequency of overall, intentional and unintentional mind wandering as a function of test type and over time in the task. Next, we look for differences in memory performance as a function of test type and attentional state.

#### Frequency of mind wandering

**Figure 1.**
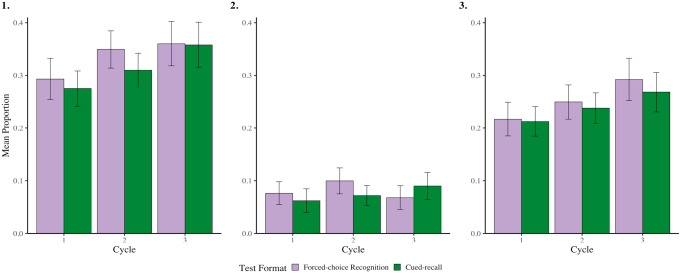
Rates of overall (panel 1), intentional (panel 2) and unintentional (panel 3) mind wandering, as a function of test format and cycle, when preparing for forced-choice recognition and cued-recall tests from Experiment 1. Error bars represent 95% CI corrected for within-subject comparisons (Morey, 2008).

Within each study-test cycle in the study phase, we calculated the proportion of probes where participants reported that they were mind wandering. We examined overall rates of mind wandering but also separated responses into intentional and unintentional mind wandering. We compared across test formats and study-test cycles using 2 (Test Format: Cued-recall vs. Forced-choice Recognition) × 3 (Cycle: 1 vs. 2 vs. 3) repeated measures analyses of variance (ANOVAs; see Figure 1 and Table 1). Although the analysis focusing on rates of unintentional mind wandering was not preregistered, we elected to include this additional analysis to provide a more complete assessment of task engagement when preparing for different test formats.

**Table 1.** Mean rates of overall, intentional, and unintentional mind wandering and media multitasking across all test formats/difficulties and cycles of Experiments 1, 2, and 3.

		Overall mind wandering							
		Easy forced-choice recognition		Difficult forced-choice recognition		Easy cued-recall		Difficult cued-recall	
		*M*	*SD*	*M*	*SD*	*M*	*SD*	*M*	*SD*
Experiment 1	Cycle 1	.293	.197	–	–	–	–	.275	.169
	Cycle 2	.349	.178	–	–	–	–	.310	.161
	Cycle 3	.360	.212	–	–	–	–	.358	.216
Experiment 2	Cycle 1	.262	.110	.284	.110	–	–	–	–
	Cycle 2	.287	.155	.248	.116	–	–	–	–
Experiment 3	Cycle 1	–	–	.221	.122	.260	.125	–	–
	Cycle 2	–	–	.285	.157	.207	.104	–	–
		Intentional mind wandering							
		Easy forced-choice recognition		Difficult forced-choice recognition		Easy cued-recall		Difficult cued-recall	
		*M*	*SD*	*M*	*SD*	*M*	*SD*	*M*	*SD*
Experiment 1	Cycle 1	.076	.110	–	–	–	–	.062	.113
	Cycle 2	.010	.123	–	–	–	–	.072	.096
	Cycle 3	.068	.114	–	–	–	–	.090	.127
Experiment 2	Cycle 1	.048	.089	.057	.100	–	–	–	–
	Cycle 2	.050	.111	.047	.092	–	–	–	–
Experiment 3	Cycle 1	–	–	.045	.089	.051	.102	–	–
	Cycle 2	–	–	.064	.118	.362	.097	–	–
		Unintentional mind wandering							
		Easy forced-choice recognition		Difficult forced-choice recognition		Easy cued-recall		Difficult cued-recall	
		*M*	*SD*	*M*	*SD*	*M*	*SD*	*M*	*SD*
Experiment 1	Cycle 1	.217	.161	–	–	–	–	.213	.142
	Cycle 2	.250	.164	–	–	–	–	.238	.145
	Cycle 3	.292	.203	–	–	–	–	.268	.188
Experiment 2	Cycle 1	.214	.104	.227	.100	–	–	–	–
	Cycle 2	.237	.128	.202	.115	–	–	–	–
Experiment 3	Cycle 1	–	–	.177	.102	.209	.124	–	–
	Cycle 2	–	–	.221	.161	.171	.108	–	–
		Media multitasking							
		Easy forced-choice recognition		Difficult forced-choice recognition		Easy cued-recall		Difficult cued-recall	
		*M*	*SD*	*M*	*SD*	*M*	*SD*	*M*	*SD*
Experiment 2	Cycle 1	.057	.114	.019	.106	–	–	–	–
	Cycle 2	.036	.108	.063	.128	–	–	–	–
Experiment 3	Cycle 1	–	–	.034	.095	.017	.107	–	–
	Cycle 2	–	–	.023	.977	.060	.121	–	–

*M* and *SD* represent mean and standard deviation, respectively.

In overall mind wandering, there was no main effect of Test Format, *F*(1,99) = 2.044, *p* = .156, *η_p_^2^* = .020, 95% CI = [.000, .100] or interaction, *F*(2,198) = .538, *p* = .585, *η_p_^2^* = .005, 95% CI = [.000, .040]. However, there was a main effect of Cycle, *F*(2,198) = 6.615, *p* = .003, *η_p_^2^* = .060, 95% CI = [.010, .130] such that rates of overall mind wandering increased from Cycle 1 to Cycle 2, *t*(99) = 2.803, *p* = .012, Cohen’s *d* = .280, 95% CI = [.080,.480], and from Cycle 1 to Cycle 3, *t*(99) = 3.219, *p* = .005, Cohen’s *d* = .320, 95% CI = [.120,.520]. Rates of overall mind wandering did not change from Cycle 2 to Cycle 3, *t*(99) = 1.326, *p* = .188, Cohen’s *d* = .13, 95% CI = [−.06, .33] (the Holm correction was used to account for multiple comparisons).

For intentional mind wandering, there were no main effects or interactions (Test Format: *F*(1,99) = .381, *p* = .539, *η_p_^2^* = .004, 95% CI = [.000, .060]; Cycle: *F*(2,198) = 1.151, *p* = .318, *η_p_^2^* = .010, 95% CI = [.000, .050]; Interaction: *F*(2,198) = 2.671, *p* = .072, *η_p_^2^* = .030, 95% CI = [.000, .080]).

For unintentional mind wandering, there was no main effect of Test Format, *F*(1,99) = 1.009, *p* = .318, *η_p_^2^* = .010, 95% CI = [.000, .080], or interaction, *F*(2,198) = .202, *p* = .817, *η_p_^2^* = .002, 95% CI = [.00, .020]. Like in overall mind wandering though, there was a main effect of Cycle, *F*(2,198) = 6.511, *p* = .003, *η_p_^2^* = .060, 95% CI = [.010, .130], such that rates of unintentional mind wandering increased from Cycle 1 to Cycle 3, t(99) = 3.211, *p* = .005, Cohen’s d = .320, 95% CI = [.120, .520] (the Holm correction was used to account for multiple comparisons). Comparisons between Cycles 1 and 2, and Cycles 2 and 3, were non-significant, all p’s > .087.

These results suggest that individuals do not strategically modify the extent to which they engage in intentional mind wandering when preparing for different test formats. However, as the amount of time-on-task increases, so does rates of (predominantly unintentional) mind wandering, replicating previous findings (Farley et al., 2013; Metcalfe & Xu, 2016; Risko et al., 2012; Thomson, Seli, et al., 2014; Wammes & Smilek, 2017).

#### Memory performance

Participants’ cued-recall and forced-choice recognition responses were scored as either correct (1) or incorrect (0). We then selected only the test trials corresponding to items immediately preceding where a thought probe had occurred during encoding. Although we preregistered a traditional ANOVA, many participants did not have data that fell into every cell of our design. For example, one participant may not have reported intentional mind wandering whatsoever. Because traditional ANOVAs handle these missing cells by removing an entire participant from the analysis, using this approach was intractable given the data. Our sample size would have been reduced by 34 participants, despite these participants providing meaningful data. As an alternative, and as recommended by others (Murayama et al., 2014), we used a logistic mixed-effects modelling approach to examine whether the relation between probe response (i.e., intentional mind wandering, unintentional mind wandering, on-task) and memory performance varied as a function of expected test format, (implemented using the *lme4* package for R; Bates et al., 2015). This approach allowed us to model the relation between memory performance and mind wandering at the level of each individual trial (see Brown, 2021 for an overview of linear mixed-effect modelling) while also maintaining our full sample size (such models can handle missing observations), as well as controlling for normative differences in stimulus difficulty. We constructed mixed-effects models consisting of by-participant and by-item random intercepts, and slopes for the effects of select predictor variables,^2^ along with fixed-effect terms capturing the overall effects of Probe Response, expected Test Format as well as their interaction. The reliability of the key fixed-effect terms was evaluated using *chi-square* tests derived from the *Anova* function from the *car* package for R (Fox & Weisberg, 2011). When interactions were present, simple effects were evaluated using the *emmeans* function from the *emmeans* package for R (Lenth, 2021). In addition, sum-to-zero coding of discrete variables was used in all analyses.

**Figure 2.**
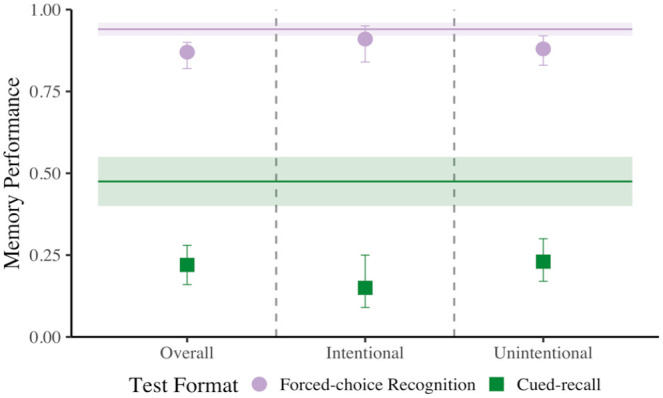
Comparison of forced-choice recognition and cued-recall memory performance when mind wandering relative to on-task in Experiment 1. These data are first plotted as overall mind wandering (i.e., collapsed across intentionality; panel 1), then subdivided into intentional (panel 2) and unintentional mind wandering (panel 3). The two horizontal line segments represent on-task memory performance for each test format, and error bars represent 95% CI.

First, using our mixed-effects models, we compared the effect of the expected test format on memory performance for items immediately preceding probe responses indicating that participants were on-task, relative to mind wandering, regardless of reported intentionality (see Figure 2, panel 1). Our mixed-effect logistic regression model (see Supplementary Material Model D1 and Table D1 for all relevant test statistics) revealed a significant effect of Probe Response where a later correct response was .595 as likely when mind wandering relative to on-task, *χ^2^*(1) = 61.892, *p* < .001, Cohen’s *d* = −.286. There was also a main effect of Test format, where recognition tests were 4.610 more likely to elicit correct responses than cued-recall tests, *χ^2^*(1) = 419.645, *p* < .001, Cohen’s *d* = .843. Surprisingly, there was no interaction between Probe Response and Test Format, *χ^2^*(1) = 1.0395, *p* = .308, Cohen’s *d* = .035. Thus, as shown in previous literature (Mooneyham & Schooler, 2013; Risko et al., 2012; Smallwood, Baracaia, et al., 2003), engaging in off-task thought generally appears to be detrimental to memory performance, and this decrement appears to hold regardless of the format and/or difficulty of the expected test format.

Next, we investigated intentional and unintentional forms of mind wandering separately, as both have been associated with reduced learning (Seli, Wammes, et al., 2015; Wammes, Boucher, et al., 2016). In intentional mind wandering (see Figure 2, panel 2; see also Supplementary Material Model D2 and Table D1 for all relevant test statistics), there was a main effect of Probe Response where a correct response was .589 as likely when intentionally mind wandering relative to on-task, *χ^2^*(1) = 23.734, *p* < .001, Cohen’s *d* = −.291. In addition, there was a main effect of Test format, where recognition tests were 5.729 more likely to elicit correct responses than cued-recall tests, *χ^2^*(1) = 236.042, *p* < .001, Cohen’s *d* = .962. These findings resonate with the findings from overall mind wandering. However here, unlike in overall mind wandering, there was a significant interaction, *χ^2^*(1) = 6.878, *p* = .009, Cohen’s *d* = .149, where probe response influenced memory performance for cued-recall tests, *z* = −5.374, *p* < .001, Cohen’s *d* = −.880, 95% CI = [−1.202, −.559], odds ratio (*OR*) *=* .203, but not forced-choice recognition tests, *z* = −1.719, *p* = .086, Cohen’s *d* = −.285, 95% CI = [−.611, .040], *OR* = .596. These results suggest that intentional mind wandering detrimentally affects memory but imposes a greater cost on free-recall than on recognition performance.

Moving on to the relation between unintentional mind wandering and memory performance (see Figure 2 panel 3; see also Supplementary Material Model D2 and Table D1 for all relevant test statistics), we found a significant effect of Probe Response, where performance was worse when unintentionally mind wandering relative to on-task, *χ^2^*(1) = 55.003, *p* < .001, Cohen’s *d* = .258, *OR* *=* .626. In addition, we found that correct answers were more likely in forced-choice recognition relative to cued-recall, *χ^2^*(1) = 500.504, *p* < .001, Cohen’s *d* = .836, *OR* *=* 4.557. Unlike in intentional mind wandering, there was no interaction, *χ^2^*(1) = 2.064, *p* = .151, Cohen’s *d* = .047. This indicates that unintentional mind wandering reduces memory performance, but the costs are relatively uniform across test types.

### Discussion

The results of Experiment 1 demonstrate that the expected test format does not seem to affect the frequency at which individuals engage in off-task thought, intentionally or unintentionally. However, although we did not observe any differences in the frequency of mind wandering, our results indicated that engagement in off-task negatively affects memory performance and, when intentional, imposes differential costs that are dependent on the test format. That is, intentional mind wandering reduced memory performance in recall but not in recognition, a pattern that did not emerge in overall or unintentional mind wandering. This suggests that while individuals’ conscious decisions to engage in off-task thought are consistent across test formats, their decisions are not calibrated to the task demands. In the “General Discussion” section, we will return to a more in-depth analysis of this finding.

Thus far, the evidence does not support the prediction that test format affects the frequency of mind wandering. In Experiment 1, we were probing for the possibility that participants would develop an expectation about the level of difficulty or the attentional requirements of a given test format and that this expectation would, in turn, lead to strategic modulation of mind wandering rates. However, this assumes that participants are in tune with the relative demands of different memory tests and can use this to inform their attentional engagement. If, however, people are not in tune with relative task demands, then they would not have the requisite knowledge to effectively calibrate or modify their mind wandering behaviour. To address this issue, we incorporated measures of participants’ confidence in their memory performance, to determine whether changes in this, or in their actual observed memory performance, predicted changes in the frequency of mind wandering.

Although we did not find that mind wandering rates differed as a function of test format, Experiment 1 did provide some evidence that intentional mind wandering differentially affected cued-recall and forced-choice recognition performance. However, although these two tests differed in format, they also differed in difficulty. With this, it remains unclear whether the differential mnemonic costs observed in Experiment 1 truly reflect differences in test format, or whether the same pattern of results would be observed when test difficulty is the only difference. Experiment 2 handles this issue by varying only the difficulty of a forced-choice recognition test and holding a constant test format.

## Experiment 2

In Experiment 2, we made three critical changes to our design from Experiment 1. First, we removed the cued-recall task and instead varied the difficulty of the forced-choice recognition task. This was done to probe whether the mnemonic cost of intentional mind wandering depends on test difficulty while holding the test format constant. Second, we sought to determine whether participants’ confidence in their learning, changes in these confidence levels, and actual memory performance predicted changes in mind wandering during a subsequent encoding phase. This was done to determine whether participants were sensitive to changes in the demands of the upcoming task, and whether this predicted mind-wandering rates. Third, we included an option for participants to indicate that they were media multitasking during encoding. This was included due to the extensive number of participants (*n* = 89) excluded from Experiment 1 for indicating they used a cellphone at some point during the experiment. In addition, including the option for participants to report media multitasking allows us to distinguish between inattention due to internal thought processes and inattention due to external distractors. Importantly, like mind wandering, when media multitasking, attentional resources are also removed from the primary task; however, such removal may be more complete than when mind wandering. In such instances, the details associated with the learning episode are likely to be poorly encoded, leaving a relatively impoverished memory representation (Castel & Craik, 2003; Jacoby et al., 1989; Jennings & Jacoby, 1993).

In our new design, participants completed two study-test cycles composed of one easy and one difficult forced-choice recognition test with different items in each cycle. As in Experiment 1, we measured rates of intentional and unintentional mind wandering in each study phase using self-report thought probes. In addition, prior to and immediately following each test phase, participants rated their confidence in memory performance (i.e., participants made both prospective and retrospective confidence judgements). Insofar as changes in rates of mind wandering are influenced by individuals’ confidence, changes in confidence, and actual memory performance, we expected that these variables might be positively correlated with changes in mind wandering rates. In addition, like Experiment 1, we explored whether the mnemonic costs of mind wandering differed based on-task demand—in this instance, whether the participant completed either an easy or a difficult forced-choice recognition test. To the extent that the observed differential cost of intentional mind wandering observed in Experiment 1 was due solely to differences in test difficulty (and not test format), this form of off-task thought should disproportionately impair memory performance for difficult forced-choice recognition memory tests.

### Method

All methods and statistical analyses were preregistered on As Predicted (aspredicted.org; reference #53906). Any analyses that were preregistered but not conducted, or any additional analyses that were not preregistered are described in text.

#### Participants

One hundred and sixty-eight participants were recruited from Prolific (2020; *Mean* age = 24.10, *SD* = 4.09, 55 female, 77 identifying as students) and received monetary compensation ($15 CND) in exchange for their participation. Participants were eligible for the study if they had English proficiency, were between the ages of 18 and 35 years of age, and had normal or corrected to normal vision. No geographic exclusions were used in participant recruitment (48% from Europe, 36% from North America, 8% from South America, 5% from Australia/Oceania, 2% from Africa, and 1% from Asia).

Our preregistered exclusion criteria were applied in the same way as in Experiment 1 (see Supplementary Material A and Experiment 2 Procedure for more detail). Seventeen participants who incorrectly responded to three or more (~40% or more) out of the six instruction questions on the first try were excluded. As well, 51 participants who indicated they wrote down word pairs during the study phase, or who indicated they left the room at some point during the study were also excluded. This left a total of 100 participants in the final sample, as intended in our preregistration. The overall exclusion rate was 40%.

#### Materials

Like Experiment 1, this experiment was programmed using PsychoPy software (Peirce et al., 2019) and was made accessible to participants through Pavlovia (2020). Participants completed the experiment online using their own personal computers and could not access the experiment on their cellphone or tablet.

Stimuli consisted of 180 weakly related cue-target word pairs (see Supplementary Material Table B3), all of which were used in Experiment 1. Participants were presented with two lists consisting of 90 word pairs each across two study-test cycles consisting of one easy and one difficult forced-choice recognition test. An independent samples t-test was conducted to ensure the mean LSA values for word pairs in each list did not differ (see Supplementary Material Table B4 for statistical output). The ordering of the two lists was randomised, as was the difficulty of the test completed in the first study-test cycle (i.e., easy or difficult). Furthermore, the order of the word pairs was randomised within each list.

#### Procedure

Prior to beginning the experiment, participants were asked to turn off their cellphone, email, and music to minimise distractions. After providing informed consent, participants were given some general instructions regarding what was required of them during the experiment and a brief definition of mind wandering (see Supplementary Material C for a detailed description of the instructions given to participants regarding mind wandering).

##### Study phase

Study phases were identical to those in Experiment 1, with only two exceptions. First, participants were only ever told that they would be preparing for a forced-choice recognition test, and the difficulty of the test was not revealed. Second, each thought probe also included the option for participants to select that they were media multitasking (i.e., using a device to view content unrelated to the task).

##### Test phase

Each test phase began with a 1 min math (distractor) task, which was identical across both study-test cycles. Following the distractor task, participants were asked to rate their confidence in their prospective memory performance from 0 to 100. Specifically, 0 would indicate that they felt they would correctly recognise none of the targets, and 100 would indicate that they felt they would correctly recognise all the targets. After making this rating, participants then completed either an easy or a difficult forced-choice recognition test.

##### Easy forced-choice recognition

The easy forced-choice recognition test was identical to the one used in Experiment 1.

##### Difficult forced-choice recognition

The difficult forced-choice recognition test was identical to the easy forced-choice recognition test, except that the lure words presented were semantically related to the cue and target words (see Supplementary Material Table B3) and therefore remained constant across word pairs and participants.

After completing either the easy or difficult forced-choice recognition test, participants were again asked to rate their confidence in their memory performance, this time retrospectively, from 0 to 100. Following this rating, participants completed a second study-test cycle wherein the forced-choice recognition test was of the remaining difficulty level (i.e., easy/difficult or difficult/easy). After completion of this final study-test cycle, participants were asked two follow-up questions as to whether they wrote anything down during either study phase to help with memory performance, or if they experienced any interruptions during the experiment (e.g., cellphone, another person in the room, leaving the experiment at any time; see Supplementary Material A). Following these questions, participants were debriefed and thanked for their participation.

### Results

#### Frequency of mind wandering

**Figure 3.**
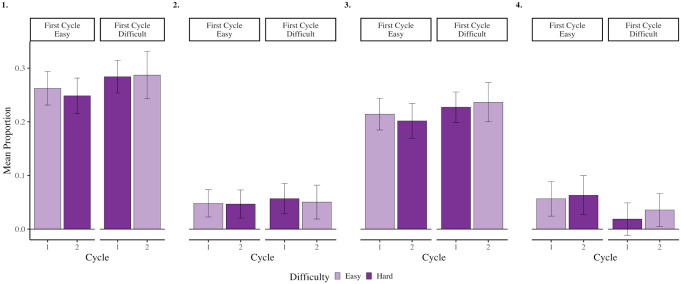
Rates of overall (panel 1), intentional (panel 2) and unintentional (panel 3) mind wandering and media multitasking (panel 4) when preparing for easy and difficult forced-choice recognition tests in Cycles 1 and 2 from Experiment 2. Error bars represent 95% CI corrected for within-subject comparisons (Morey, 2008).

Although not preregistered, to be consistent with Experiment 1, we compared rates of overall, intentional and unintentional mind wandering and media multitasking when preparing for an easy and difficult forced-choice recognition test using four paired-samples *t*-tests. All analyses were non-significant, all *t*’s < .781, and all *p*’s > .436. This result is not surprising, given that we did not inform participants of the difficulty of the upcoming test. More relevant to our primary research question, we found that the change in overall, intentional and unintentional mind wandering and media multitasking from Cycle 1 to Cycle 2 did not differ depending on whether participants completed a difficult or easy forced-choice recognition test in the first cycle, all *F*’s < .604, and all *p*’s > .438, (see Figure 3, and Table 1).^3^

#### Confidence in memory performance

To validate our manipulation of test difficulty and assess whether participants' confidence ratings were accurate, we used a 2 (Rating Time: Before vs. After) x 2 (Test Difficulty: Easy vs. Difficult) repeated measures ANOVA to compare mean confidence ratings provided before and after easy and difficult forced-choice recognition tests. There was no main effect of Rating Time, *F*(1,99) = .064, *p* = .800, *η_p_^2^* = .001, 95% CI = [.000, .040]. In contrast, there was a main effect of Difficulty, *F*(1,99) = 5.029, *p* = .027, *η_p_^2^* = .050, 95% CI = [.000, .150], as well as an interaction between Difficulty and Rating Time, *F*(1,99) = 19.984, *p* < .001, *η_p_^2^* = .170, 95% CI = [.060, .300]. This interaction was driven by an increase in confidence ratings from before (*M* = 50.160, 20.492) to after the test (*M* = 54.810, *SD* = 22.195), *t*(99) = 2.760, *p* = .007, Cohen’s *d* = .280, 95% CI = [.080, .480] when it was easy, and a decrease (before: *M* = 52.150, *SD* = 21.031; after: *M* = 46.860, *SD* = 20.591) when it was difficult, *t*(99) = 3.152, *p* = .002, Cohen’s *d* = .320, 95% CI = [.110, .520]. This indicates that our manipulation successfully created subjective differences in difficulty for the two forced-choice recognition tests.

As outlined, one of the major changes made in this Experiment was the inclusion of confidence ratings to assay whether rates of mind wandering or media multitasking could be predicted by this measure. However, our analyses indicated that confidence ratings, changes in these ratings, and observed memory performance did not predict changes in the rates of any form of off-task thinking, all |*r|*’s < .183, and *p*’s > .068 (see Table 2 for all relevant test statistics).

**Table 2.** All relevant test statistics for correlations between participants’ confidence in memory performance, change in confidence, and actual memory performance (cycle 1), and participants’ change in overall, intentional and unintentional mind wandering, and media multitasking in Experiments 2 and 3.

Change in:	Experiment 2					
	Confidence in memory performance		Change in confidence		Actual memory performance	
	*r* [95% CI]	*p*	*r* [95% CI]	*p*	*r* [95% CI]	*p*
Overall mind wandering	–.047 [–.241, .151]	.641	–.054 [–.248, .144]	.595	–.026 [–.222, .171]	.795
Intentional	–.052 [–.246,.146]	.606	–.034 [–.228,.164]	.740	–.025 [–.220, .172]	.805
Unintentional	–.002 [–.198, .195]	.985	–.045 [–.240,.152]	.654	–.008 [–.204, .189]	.938
Media multitasking	–.022 [–.218, .175]	.827	–.183 [–.366, .014]	.069	–.135 [–.323, .063]	.180
Change in:	Experiment 3					
	Confidence in-memory performance		Change in confidence		Actual memory performance	
	*r* [95% CI]	*p*	*r* [95% CI]	*p*	*r* [95% CI]	*p*
Overall mind wandering	–.053 [–.247, .145]	.598	–.076 [–.268, .123]	.454	–.010 [–.206, .187]	.925
Intentional	–.068 [–.261, .130]	.502	–.039 [–.234, .159]	.701	–.053 [–.246, .145]	.604
Unintentional	–.010 [–.187, .206]	.921	–.079 [–.271, .119]	.434	.068 [–.131, .261]	.504
Media multitasking	–.098 [–.289, .100]	.330	.060 [–.138, .254]	.550	–.071 [–.264, .127]	.480

CI: Confidence interval.

#### Memory performance

Similar to Experiment 1, participants' forced-choice recognition responses were scored as either correct (1) or incorrect (0), and only test trials corresponding to items immediately preceding where a thought probe had occurred during encoding were selected. Again, we analysed whether the relation between probe response (i.e., intentional mind wandering, unintentional mind wandering, media multitasking, on-task) and memory performance varied as a function of test difficulty. Similar to Experiment 1, although we preregistered these analyses as traditional ANOVAs, many participants did not use each of the probe response options. Therefore, we used a logistic mixed-effects modelling approach which allowed us to maintain our full sample size, and to control for item-level differences in memorability. Again, we constructed mixed-effects models consisting of by-participant and by-item random intercepts and slopes for the effects of select predictor variables, along with fixed-effect terms capturing the overall effects of probe response, test difficulty, and their interaction.

**Figure 4.**
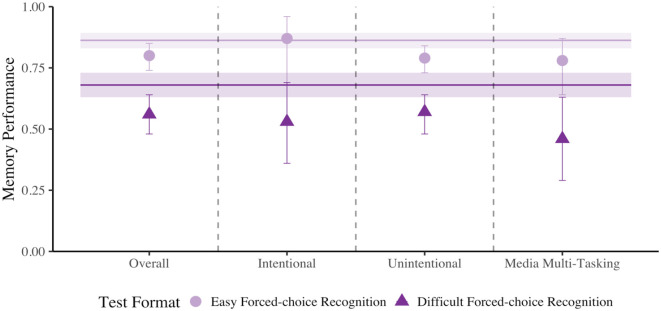
Comparison of easy and difficult forced-choice recognition memory performance when off-task relative to on-task in Experiment 2. Data are first plotted as overall mind wandering (panel 1), then intentional (panel 2) and unintentional mind wandering (panel 3), then media multitasking (panel 4). The two horizontal line segments represent on-task memory performance for each test difficulty, and error bars represent 95% CI.

First, using our mixed-effects models, we compared memory performance for items immediately preceding probe responses indicating that participants were on-task and those that indicated they were mind wandering, regardless of intentionality (see Figure 4, panel 1). Our mixed-effect logistic regression model (see Supplementary Material Model D3 and Table D2 for all relevant test statistics) revealed significant effects of Probe Response where a later correct response was .791 as likely when mind wandering relative to on-task, *χ^2^*(1) = 14.156, *p* < .001, Cohen’s *d* = .129. There was also a main effect of Test Difficulty where easy forced-choice recognition tests were 1.747 more likely to elicit correct responses than difficult forced-choice recognition tests, *χ^2^*(1) = 90.756, *p* < .001, Cohen’s *d* = .308. Similar to Experiment 1, we did not observe a significant interaction between Probe Response and Test Difficulty, *χ^2^*(1) = .104, *p* = .747, Cohen’s *d* = .010.

Next, we investigated intentional and unintentional mind wandering separately. For intentional mind wandering (see Figure 4, panel 2; see also Supplementary Material Model D4 and Table D2 for all relevant test statistics) there was a significant main effect of Test Difficulty, where easy forced-choice recognition tests were 2.063 more likely to elicit a correct response than difficult forced-choice recognition tests, *χ^2^*(1) = 18.968, *p* < .001, Cohen’s *d* = .399. Interestingly, the effect of Probe Response was not significant, *χ^2^*(1) = .767, *p* = .381, Cohen’s *d* = -.087, nor was the interaction between Probe Response and Test Difficulty, *χ^2^*(1) = 1.034, *p* = .309, Cohen’s *d* = .093.

Next, for the relation between unintentional mind wandering and memory performance (see Figure 4, panel 3; see also Supplementary Material Model D3 and Table D2 for all relevant test statistics), we found a significant effect of Probe Response, where performance was worse when unintentionally mind wandering relative to on-task, *χ^2^*(1) = 13.484, *p* < .001, Cohen’s *d* = -.133, *OR* = .786. In addition, we found that correct responses were more likely in easy forced-choice recognition relative to difficult forced-choice recognition tests, *χ^2^*(1) = 75.265, *p* < .001, Cohen’s *d* = .297, *OR* = 1.714. The interaction between Probe Response and Test Difficulty was not significant, *χ^2^*(1) = .0002, *p* = .989, Cohen’s *d* = .0005.

Last, we explored the relationship between media multitasking and memory performance (see Figure 4, panel 4; see also Supplementary Material Model D3 and Table D2 for all relevant test statistics). This analysis revealed a significant effect of Probe Response, where a correct response was .684 as likely when media multitasking relative to on-task, *χ^2^*(1) = 8.422, *p* = .004, Cohen’s *d* = -.209. We also observed a significant effect of Test Difficulty, where easy forced-choice recognition tests were 1.875 more likely to elicit correct responses than difficult forced-choice recognition tests, *χ^2^*(1) = 26.351, *p* < .001, Cohen’s *d* = .346. The interaction between Probe Response and Test Difficulty was not significant, *χ^2^*(1) = .491, *p* = .483, Cohen’s *d* = .047.

### Discussion

The results of Experiment 2 demonstrate that confidence and changes in confidence in memory performance and actual memory performance did not predict changes in the frequency of off-task thought as a function of test difficulty. More specifically, although participants’ confidence in memory performance was closely aligned with actual memory performance, neither was correlated with changes in the frequency of overall, intentional or unintentional mind wandering or media multitasking. This finding, or lack thereof, coupled with those from Experiment 1 suggests that engagement in off-task thought is insensitive to these specific conditions of learning (e.g., test format, test performance, and memory confidence).

An interesting outcome from Experiment 2 was that we observed no performance deficits in forced-choice recognition memory associated with intentional mind wandering regardless of test difficulty. To reconcile this with Experiment 1, where there *was* a greater cost of intentional mind wandering in recall tasks, one possibility is that intentional mind wandering is titrated to differences in test format, not necessarily to test difficulty. An alternative possibility is that, although overall memory performance was reduced in the difficult relative to the easy forced-choice recognition condition, the increase in difficulty was insufficient to observe a greater cost of mind wandering on memory performance. It is also possible that the absence of a main effect of Probe Response when considering intentional cases of mind wandering may simply reflect reduced power relative to Experiment 1 as there were fewer study-test cycles. Therefore fewer instances of intentional mind wandering in Experiment 2. The reduced number of off-task probe responses may also have lowered our power to detect a small interaction between Probe Response and Test Difficulty when considering intentional cases of mind wandering. We address these concerns in a final combined analysis following Experiment 3.

In Experiment 1, we tested whether anticipation of a more difficult test format would lead to reduced mind wandering, but instead found that although mind wandering rates did not differ, the *costs* of mind wandering differed, at least when it was intentional. However, it was unclear whether this finding was due to differences in the test format itself, or simply due to the fact cued-recall is more difficult than forced-choice recognition. In Experiment 2, we aimed to address this issue by keeping the test format constant and titrating difficulty. We found no differential costs of mind wandering as a function of test difficulty. In Experiment 3, we aimed to do the reverse, keeping difficulty relatively constant but changing the test format. Doing so would provide some more concrete answers about whether it is task difficulty, test format, or some combination of the two that predicts the costs of mind wandering.

## Experiment 3

The general structure of Experiment 3 was similar to Experiment 2, with the only exception being that instead of holding the test format constant and manipulating difficulty, we attempted to equate difficulty and manipulate the test format. To do this, we used the difficult forced-choice recognition test from Experiment 2 and made the cued-recall test from Experiment 1 easier by providing participants with the cue and the first two letters of each target word as a prompt for recall (instead of just the cue). To the extent that differences in test format, rather than test difficulty, are responsible for differential mnemonic costs associated with intentional mind wandering, we expected that intentional mind wandering would negatively affect memory performance on cued-recall, but not forced-choice recognition tests.

### Method

All methods and statistical analyses were preregistered on As Predicted (aspredicted.org; reference #59523). Any analyses that were preregistered but not conducted or any additional analyses that were not preregistered are described in text.

#### Participants

One hundred and twenty-four participants were recruited from Prolific (2020; *Mean* age = 25.09, *SD* = 4.27, 45 female, 68 identifying as students) and received monetary compensation ($15 CND) in exchange for their participation. Participants were eligible for the study if they had English proficiency, were between the ages of 18 and 35, and had normal or corrected to normal vision. No geographic exclusions were used in participant recruitment (46% from North America, 41% from Europe, 6% from South America, 2% from Australia/Oceania, Africa and Asia, and 1% unknown).

Our preregistered exclusion criteria were applied in the same way as in Experiments 1 and 2 (see Supplementary Material A and Experiment 3 Procedure for more detail). Twelve participants who incorrectly responded to four or more (~40% or more) out of the nine instruction questions on the first try were excluded. Also, 12 participants who indicated they wrote down word pairs during the study phase, or who indicated they left the room at some point during the study were also excluded. This left a total of 100 participants in the final sample, as intended in our preregistration. The overall exclusion rate was 19%.

#### Materials

Similar to Experiments 1 and 2, this experiment was programmed using PsychoPy software (Peirce et al., 2019) and was made accessible to participants through Pavlovia (2020). Participants completed the experiment online using their own personal computers and were unable to access the experiment on their cellphone or tablet.

Stimuli were the same 180 weakly related cue-target word pairs used in Experiment 2 (see Supplementary Material Table B3). Participants were presented with two lists consisting of 90 word pairs each across two study-test cycles consisting of one difficult forced-choice recognition test and one easy cued-recall test. Ordering of the two lists was randomised, as was the test format completed in the first study-test cycle (i.e., difficult forced-choice recognition or easy cued-recall). Furthermore, the order of the word pairs was randomised within each list.

#### Procedure

Prior to beginning the experiment, participants were asked to turn off their cellphone, email, and music to minimise distractions. After providing informed consent, participants were given general instructions regarding what was required of them during the experiment as well as a brief definition of mind wandering (see Supplementary Material C for a detailed description of the instructions given to participants regarding mind wandering).

##### Study phase

Study phases were identical to those in Experiment 1, with the only exception being that each thought probe also included the option for participants to select that they were media multitasking (i.e., using a device to view content unrelated to the task).

##### Test phase

Participants completed the same distractor task and confidence ratings as in Experiment 2. After providing pretest confidence ratings, participants completed either a difficult forced-choice recognition test or an easy cued-recall test.

##### Difficult forced-choice recognition

The difficult forced-choice recognition test was identical to the one in Experiment 2.

##### Easy cued-recall

The easy cued-recall test was identical to the one used in Experiment 1, with only one distinction. Specifically, on each trial, the first two letters of the target word were presented above the textbox provided. This was done to help participants generate the target word.

After completion of either the difficult forced-choice recognition test or the easy cued-recall test, participants provided retrospective confidence ratings (as in Experiment 2). Following this rating, participants had completed one study-test cycle. Participants then completed an additional study-test cycle consisting of the alternative test format. After completion of the final study-test cycle, participants were asked two follow-up questions as to whether they wrote anything down during either study phase to help with memory performance, or if they experienced any interruptions during the experiment (e.g., cellphone, another person in the room, leaving the experiment at any time; see Supplementary Material A). Following these questions, participants were debriefed and thanked for their participation.

### Results

#### Frequency of mind wandering

**Figure 5.**
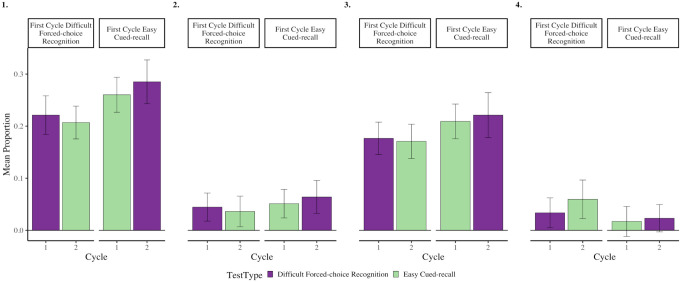
Rates of overall (panel 1), intentional (panel 2) and unintentional (panel 3) mind wandering and media multitasking (panel 4) when preparing for difficult forced-choice recognition and easy cued-recall tests from Experiment 3. Error bars represent 95% CI corrected for within-subject comparisons (Morey, 2008).

Although not preregistered, to be consistent with prior Experiments, we compared rates of overall, intentional and unintentional mind wandering and media multitasking when preparing for a difficult forced-choice recognition test and an easy cued-recall test using four paired-sample *t*-tests. All analyses were non-significant, all *t*’s < 1.087, and all *p*’s > .280. Again, this is not surprising given that we observed no difference in the rates of mind wandering in Experiment 1. In addition, we found that the change in overall, intentional and unintentional mind wandering, and media multitasking from Cycle 1 to Cycle 2 did not differ depending on whether participants completed a difficult forced-choice recognition or easy cued-recall test in the first cycle, all *F*’s < 1.097, and all *p*’s > .296 (see Figure 5, and Table 1).^4^

#### Confidence in memory performance

To appraise the match in test difficulty between test formats and assess whether participants’ confidence ratings were both accurate and similar across test formats, we conducted a 2 (Rating Time: Before vs. After) × 2 (Test Format: Difficult Forced-choice Recognition vs. Easy Cued-recall) repeated measures ANOVA, comparing mean confidence ratings provided before and after each test. We observed significant main effects of Rating Time, *F*(1,99) = 6.065, *p* = .016, *η_p_^2^* = .060, 95% CI = [.000, .170], and Test Format, *F*(1,99) = 32.320, *p* < .001, *η_p_^2^* = .250, 95% CI = [.110, .380]. As well, the interaction between Test Format and Rating Time was significant, *F*(1,99) = 63.641, *p* < .001, *η_p_^2^* = .390, 95% CI = [.250, .510]. Similar to Experiment 2, this interaction was driven by an increase in confidence rating from before (*M* = 43.40, *SD* = 24.272) to after the test (*M* = 48.490, *SD* = 24.998), *t*(99) = 3.036, *p* = .003, Cohen’s *d* = .310, 95% CI = [.100, .510] when the expected test format was difficult forced-choice recognition, and a decrease (before: *M* = 42.770, *SD* = 21.215; after: *M* = 30.670, *SD* = 22.085) when the expected test format was easy cued-recall, *t*(99) = 6.412, *p* < .001, Cohen’s *d* = .640, 95% CI = [.430, .860]. Thus, despite our attempt to make the cued-recall test easier by providing the first two letters of each target word, participants nevertheless perceived these tests to be more challenging than the forced-choice recognition test with similar lure items.

Much like in Experiment 2, our analyses indicated that confidence ratings, changes in these ratings, and observed memory performance did not predict changes in the rates of any form of off-task thinking, all |*r|*’s < .098, and *p*’s > .330 (see Table 2 for all relevant test statistics).

#### Memory performance

**Figure 6.**
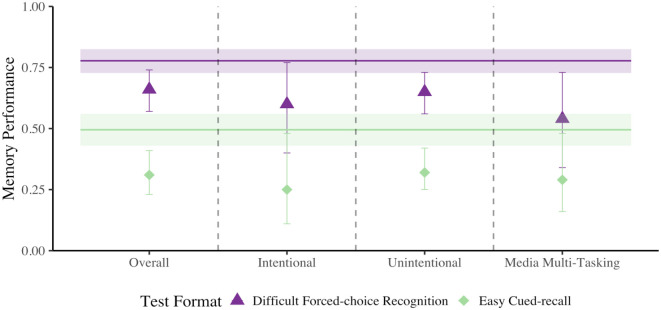
Comparison of difficult forced-choice recognition and easy cued-recall memory performance when off-task relative to on-task in Experiment 3. Data are first plotted as overall mind wandering (panel 1), then intentional (panel 2) and unintentional (panel 3) mind wandering, then media multitasking (panel 4). The two horizontal line segments represent on-task memory performance for each test format, and error bars represent 95% CI.

Our logistic mixed-effects modelling approach here was identical to Experiments 1 and 2. First, using our mixed-effects models, we compared the effect of the expected test format on memory performance for items immediately preceding probe responses indicating that participants were on-task and those that indicated they were mind wandering, regardless of intentionality (see Figure 6, panel 1). Our mixed-effect logistic regression model (see Supplementary Material Model D5 and Table D3 for all relevant test statistics) revealed a significant effect of Probe Response where a later correct response was .709 as likely when mind wandering relative to on-task, *χ^2^*(1) = 21.148, *p* < .001, Cohen’s *d* = −.190. There was also a main effect of Test Format where difficult forced-choice recognition tests were 1.977 more likely to elicit correct responses than cued-recall tests, *χ^2^*(1) = 89.892, *p* < .001, Cohen’s *d* = .376. Similar to Experiments 1 and 2, we did not observe a significant interaction between Probe Response and Test Format, *χ^2^*(1) = .346, *p* = .557, Cohen’s *d* = .021.

Next, we investigated intentional and unintentional mind wandering separately. For intentional mind wandering (see Figure 6, panel 2; see also Supplementary Material Model D5 and Table D3 for all relevant test statistics), there was a significant effect of Probe Response where a correct response was .620 as likely when intentionally mind wandering relative to on-task, *χ^2^*(1) = 6.918, *p* = .009, Cohen’s *d* = −.264. In addition, there was a main effect of Test Format where difficult forced-choice recognition tests were 1.992 more likely to elicit correct responses than easy cued-recall tests, *χ^2^*(1) = 20.597, *p* < .001, Cohen’s *d* = .380. As in Experiment 2, the interaction between Probe Response and Test Format was not significant, *χ^2^*(1) = .085, *p* = .771, Cohen’s *d* = .024.

Moving on to the relation between unintentional mind wandering and memory performance (see Figure 6, panel 3; see also Supplementary Material Model D5 and Table D3 for all relevant test statistics), we found a significant effect of Probe Response, where performance was worse when unintentionally mind wandering relative to on-task, *χ^2^*(1) = 24.870, *p* < .001, Cohen’s *d* = −.187, *OR* = .712. In addition, we found that correct responses were more likely on a difficult forced-choice recognition relative to an easy cued-recall test, *χ^2^*(1) = 80.104, *p* < .001, Cohen’s *d* = .364, *OR* = 1.935. As in Experiments 1 and 2, the interaction between Probe Response and Test Format was not significant, *χ^2^*(1) = .088, *p* = .767, Cohen’s *d* = .011.

Finally, for media multitasking (see Figure 6, panel 4; see also Supplementary Material Model D6 and Table D3 for all relevant test statistics), our analysis again revealed a significant effect of Probe Response, where performance was worse when media multitasking relative to on-task, *χ^2^*(1) = 9.648, *p* = .002, Cohen’s *d* = −.266, *OR* = .617. In addition, we found that correct answers were more likely on a difficult forced-choice recognition relative to an easy cued-recall test, *χ^2^*(1) = 14.880, *p* < .001, Cohen’s *d* = .315, *OR* = 1.772. The interaction between Probe Response and Test Format was not significant, *χ^2^*(1) = .095, *p* = .757, Cohen’s *d* = −.025.

### Discussion

In this experiment, we had aimed to equate the difficulty of the forced-choice recognition and cued-recall tests, to discern whether there were greater costs of mind wandering in cued-recall. Although we were not successful in this goal, we nevertheless expected to observe greater costs of mind wandering for the easy cued-recall test, given that such a test provides less retrieval support than does the hard forced-choice recognition test. Such a finding would have been consistent with our results in Experiment 1, where intentional mind wandering led to greater costs to cued-recall than recognition. However, contrary to this prediction, the results of Experiment 3 revealed no evidence of a differential cost of mind wandering (regardless of intentionality) or media multitasking across test formats.

However, we found that all forms of mind wandering and media multitasking were associated with impaired subsequent memory performance relative to being on-task. This finding is completely consistent with the first two experiments. Across all the experiments, there was only one instance where the costs of mind wandering were reliably different across test formats or levels of difficulty (Experiment 1). Taken together, the foregoing findings raise two possibilities. First, it could be the case that inattention (regardless of intentionality) during encoding impairs the formation of sufficiently detailed memory traces that support memory performance for all test formats and levels of difficulty. Second, it could be the case that more strategic forms of inattention do not hamper memory performance when gist-level memory representations are sufficient for later retrieval (e.g., in memory tests that rely solely on item-based information). To adjudicate between these possibilities, and to shed further light on the conditions under which mind wandering impairs memory, we now turn to a combined analysis of the data from all experiments.

## Combined analyses

Across the three experiments reported here, we have shown (1) consistent costs of mind wandering to memory performance across various test formats and levels of difficulty and (2) that the mnemonic cost of mind wandering may be different when preparing for different kinds of tests, at least in some cases. Specifically, in Experiment 1, in which the difference in difficulty between the two test formats was greatest, the cost of intentionally mind wandering was only present for cued-recall compared to forced-choice recognition tests. Interestingly, however, this differential cost of intentional mind wandering was not reliably observed for the different test formats and difficulty levels used in Experiments 2 and 3. With this, we aimed to better understand the overarching association between task demands and the costs of mind wandering to memory performance as a function of these demands. To examine this comprehensively, we compared the mnemonic costs of mind wandering across all experiments, test formats, and test difficulties. That is, we combined data from all experiments reported here and analysed whether the relation between probe response (i.e., overall mind wandering, intentional mind wandering, unintentional mind wandering, media multitasking, on-task) and memory performance varied as a function of test difficulty (i.e., easy forced-choice recognition, difficult forced-choice recognition, easy cued-recall, difficult cued-recall), using a logistic mixed-effects modelling approach. All models were created using the same parameters described previously, and the effect of test format on the relation between probe response and memory performance was analysed separately for overall, intentional and unintentional mind wandering and media multitasking. However, it is important to note that not every test format was used in every study. Therefore, there are some instances in which the test format is a within-subjects factor and some in which it is a between-subjects factor. For this reason, in all cross-experimental analyses, the test format was treated as a between-subjects factor and was never included as a random slope in our models. These analyses were not preregistered.

### Overall mind wandering

**Figure 7.**
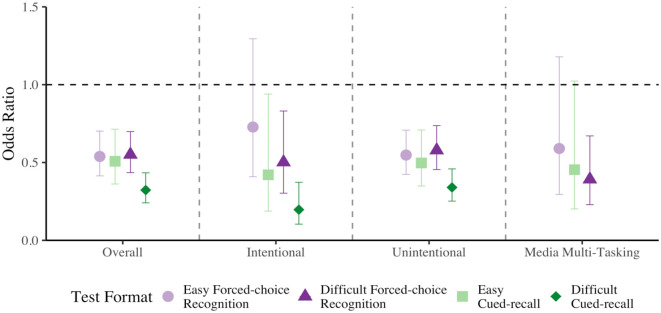
Odds ratios representing the comparison of easy and difficult forced-choice recognition, and easy and difficult cued-recall memory performance when off-task (panel 1), intentionally mind wandering (panel 2), unintentionally mind wandering (panel 3), and media multitasking (panel 4), relative to on-task, collapsed across all experiments. Errors bars represent 95% CI.

First, using our mixed-effects models, we compared memory performance for items immediately preceding probe responses indicating that participants were on-task and those that indicated they were mind wandering, regardless of intentionality (see Figure 7, panel 1). Our mixed-effect logistic regression model (see Supplementary Material Model D7) revealed significant effects of Probe Response, *χ^2^*(1) = 99.119, *p* < .001, and Test Format, *χ^2^*(1) = 869.686, *p* < .001. Critically, we also observed a significant interaction between Probe Response and Test Format, *χ^2^*(1) = 8.814, *p* = .032. Further investigation of this interaction revealed significant effects of Probe Response for both easy, *z* = −4.591, *p* < .001, Cohen’s *d* = −.341, 95% CI = [−.486, −.195], and difficult, *z* = −4.929, *p* < .001, Cohen’s *d* = −.328, 95% CI = [−.459, −.197], forced-choice recognition tests, as well as both easy, *z* = −3.898, *p* < .001, Cohen’s *d* = −.373, 95% CI = [−.560, −.186], and difficult cued-recall, *z* = −7.511, *p* < .001, Cohen’s *d* = −622, 95% CI = [−.785, −.460] (see Supplementary Material Table D4 for all relevant simple effect test statistics). Interestingly, although there appears to be a cost of mind wandering regardless of test format or difficulty, the magnitude of the cost to memory performance seems to increase as the test increases in difficulty.

### Intentional mind wandering

Looking at the relation between intentional mind wandering and memory performance, our logistic regression model (see Supplementary Material Model D7) revealed significant effects of Probe Response, *χ^2^*(1) = 27.592, *p* < .001, and Test Format, *χ^2^*(1) = 220.843, *p* < .001. Similar to the analysis of overall mind wandering, the interaction between Probe Response and Test Format was also significant, *χ^2^*(1) = 8.4268, *p* = .038. Further investigation of this interaction revealed significant effects of Probe Response for both easy, *z* = −2.110, *p* = .035, Cohen’s *d* = −.477, 95% CI = [−.921, −.034], and difficult cued-recall tests, *z* = −4.983, *p* < .001, Cohen’s *d* = −.896, 95% CI = [−1.248, −.544], as well as difficult forced-choice recognition tests, *z* = −2.681, *p* = .007, Cohen’s *d* = −.380, 95% CI = [−.658, −.102]. The effect of Probe Response on easy forced-choice recognition performance was not significant, *z* = −1.081, *p* = .280, Cohen’s *d* = −.175, 95% CI = [−.493, .143] (see Figure 7, panel 2; see also Supplementary Material Table D4 for all relevant simple effect test statistics). Thus, it appears that memory performance is *only* spared from the costs of intentionally engaging in off-task thought in the easiest test format. Here as well, the costs of mind wandering appear to scale with the difficulty of the task.

### Unintentional mind wandering

Next, for the relation between unintentional mind wandering and memory performance, our logistic regression model (see Supplementary Material Model D8) revealed significant effects of Probe Response, *χ^2^*(1) = 91.704, *p* < .001, and Test Format, *χ^2^*(1) = 759.690, *p* < .001. As well, like the analyses for overall and intentional mind wandering, the interaction between Probe Response and Test Format was also significant, *χ^2^*(1) = 8.366, *p* = .039. Further investigation of this interaction revealed significant effects of Probe Response for both easy, *z* = −4.589, *p* < .001, Cohen’s *d* = −.332, 95% CI = [−.473, −.190], and difficult, *z* = −4.429, *p* < .001, Cohen’s *d* = −.301, 95% CI = [−.434, −.168], forced-choice recognition tests, as well as both easy, *z* = −3.866, *p* < .001, Cohen’s *d* = −.385, 95% CI = [−.580, −.190], and difficult, *z* = −7.041, *p* < .001, Cohen’s *d* = −.595, 95% CI = [−.760, −.429] cued-recall (see Figure 7, panel 3; see also Supplementary Material Table D4 for all relevant simple effect test statistics). Therefore, similar to the effect of overall mind wandering, it appears that there is a cost of mind wandering regardless of test format or difficulty; however, the magnitude of the cost to memory performance seems to increase as the test increases in difficulty.

### Media multitasking

For the relation between media multitasking and memory performance, we were only able to compare performance on easy and difficult forced-choice recognition tests and difficult cued-recall tests, given that this response option was not included in Experiment 1. The analysis of our logistic regression model (see Supplementary Material Model D8) again revealed significant effects of Probe Response, *χ^2^*(1) = 13.186, *p* < .001, and Test Format, *χ^2^*(1) = 62.897, *p* < .001. However, unlike the analyses for overall, intentional, and unintentional mind wandering, the interaction between Probe Response and Test Format was not significant, *χ^2^*(1) = .884, *p* = .643. Thus, it appears that engaging in media multitasking is equally as costly to memory performance regardless of test format or difficulty (see Figure 7, panel 4; see also Supplementary Material Table D4 for all relevant simple effect test statistics).

## General discussion

The results of the current research provide interesting and novel findings that contribute to existing research on the interplay between mind wandering and memory. First, all experiments reported here demonstrate that individuals appear not to strategically regulate the extent of mind wandering depending on the format or difficulty of an upcoming test. This was the case even when participants were made aware of the specific retrieval demands required of the test and were given the opportunity to predict and reflect upon their memory performance by making proactive and retroactive confidence ratings. Second, the results indicate that any form of off-task thought almost invariably impairs later memory performance. To qualify this, the results of our combined analyses reveal that the mnemonic cost of off-task thought during encoding may scale with the difficulty of the upcoming test. Specifically, we found that for both overall and unintentional mind wandering, the memory decrement observed when participants reported being off-task as compared with on-task increased as the test increased in difficulty. Interestingly, for intentional mind wandering, although a similar scaling was observed for the three hardest test formats (i.e., difficult forced-choice recognition, easy cued-recall, and difficult cued-recall), we observed no associated memory deficit when participants completed an easy forced-choice recognition test. Importantly, this may provide some potential evidence suggesting that intentional and unintentional mind wandering may differentially affect future memory performance. Media multitasking, however, seemed to negatively affect performance uniformly across different levels of difficulty, suggesting that external distractions are much more costly than internal distractions.

### Why don’t individuals strategically regulate off-task thought?

As previously discussed, we predicted that individuals would differentially engage in off-task thought during encoding depending on the anticipated demands of an upcoming test, as previous research has shown that such strategic engagement in off-task thought can be contingent on the demands and context of the task or encoding episode (Maillet et al., 2017; Metcalfe & Xu, 2016; Seli, Carriere, et al., 2018; Seli, Konishi, et al., 2018; Thomson, Smilek, & Besner, 2014; Wammes & Smilek, 2017). We reasoned that when preparing for a relatively easy test that represents previously seen information (e.g., forced-choice recognition), individuals may be more inclined to mind wander relative to when preparing for a relatively difficult test that is generative in nature (e.g., cued-recall). However, contrary to our predictions, the results of the current research found no evidence that individuals regulate the frequency off-task thought in response to the demands of the upcoming test. However, the question remains, why don’t individuals strategically adapt their mind wandering?

One might speculate that individuals were failing to accurately monitor their test performance resulting in the perception that all tests are equally difficult. In this case, it is unlikely that differences in expected test format or difficulty would induce strategic adjustments in the frequency off-task thought. In Experiments 2 and 3, participants’ confidence ratings scaled with the difficulty of the test, meaning that not all tests were perceived as equal in difficulty. As such, during the encoding phase of the subsequent study-test cycle, participants could have relied on their performance assessment on the previous test in addition to the foreknowledge of the upcoming test format to regulate the extent of mind wandering. However, such an adjustment did not occur, as confidence ratings did not predict individuals’ engagement in, or change in engagement in off-task thought during the second encoding phase. Clearly, the failure to regulate the frequency of mind wandering as a function of the expected test format is not driven by inaccurate perceptions of test difficulty.

Alternatively, unlike in-the-moment encoding demands, it may be that the future demands of an upcoming test are simply too far removed from the current learning episode for individuals to incorporate this knowledge in regulating their attention. To our knowledge, almost all previous research examining individuals’ ability to strategically regulate mind-wandering manipulated task demands concurrent with assessments of off-task thought (Metcalfe & Xu, 2016; Seli, Carriere, et al., 2018; Seli, Konishi, et al., 2018; Wammes & Smilek, 2017). Therefore, it could be the case that participants only engage in the strategic regulation of attention when it is immediately relevant to the performance of an ongoing task. In the experiments reported here, the nominal task demands during the time at which thought probes were administered (i.e., encoding phase) did not differ across conditions. As such, participants may have discounted the demands of the upcoming test when regulating their off-task thought.

Another possibility is that learners *can* differentially regulate attention in response to upcoming test demands but that they simply did not do so under the conditions tested here, or that only certain individuals engage in this strategic titration of attention. For example, the explicit provision of additional information regarding how disengagement during encoding differentially affects memory for different tests might trigger the strategic regulation of mind wandering during encoding. Moreover, other manipulations that increase motivation to attain high levels of memory performance (e.g., points awarded for correct responses) may also induce participants to adjust the frequency of mind wandering. Finally, there is also some evidence that differences in working memory capacity predict the strategic regulation of attention (Robison & Unsworth, 2017; Rummel & Boywitt, 2014). Without a measure of working memory capacity, it is difficult to ascertain whether such individual differences contributed to the patterns of mind wandering reported here. Irrespective of these possibilities, our results imply that, at minimum, learners do not easily, consistently, or spontaneously alter the frequency of mind wandering based on the expected test format.

### Memory representations when engaged and not engaged in mind wandering

A key finding in the current study was that the costs associated with mind wandering differed depending on the test format/difficulty. One of the most obvious reasons for this differential cost may have to do with the specific memory representation encoded when individuals are and are not engaged in mind wandering. Specifically, several theories hold that when mind wandering, attentional or executive control resources that would otherwise be devoted to the current task (i.e., encoding) are reallocated to internal thought processes (Smallwood & Schooler, 2006; Thomson et al., 2015). In media multitasking, though unlikely to be driven by the same mechanism, the outcome is comparable: Attentional resources are removed from the primary task in both mind wandering and media multitasking, and in the case of media multitasking, they may be removed from the current task more completely. In the absence of outward attention directed towards the specific encoding task, the details associated with the learning episode are likely to be poorly encoded, leaving a relatively impoverished memory representation (Castel & Craik, 2003; Jacoby et al., 1989; Jennings & Jacoby, 1993). Given that different test formats/difficulties require different levels of memory representations, it is not surprising that performance on these tests is differentially affected by mind wandering during encoding. That is, when a test requires the memory representation of a learning episode to be detailed and precise (e.g., cued-recall), mind wandering may be more detrimental to memory performance. However, when the test requires only a vague sense of familiarity or gist-level memory representation (e.g., forced-choice recognition), mind wandering may not be as detrimental because any residual attentional resources directed towards encoding may be sufficient to create a gist-based or familiarity-based memory representation. Although this reasoning seems quite clear, why do we see different patterns of results depending on the intentionality with which individuals engage in off-task thought (i.e., intentional vs. unintentional)?

#### Mnemonic costs of mind wandering: all-or-none or graded?

As previously mentioned, the results of the combined analysis revealed that the mnemonic cost of mind wandering may scale with the difficulty of the upcoming test. Specifically, for unintentional mind wandering, a memory decrement was observed when participants reported being off-task as compared with on-task regardless of the format/difficulty of the memory test. Notably, the cost also increased with increasing test difficulty (see Figure 7, panel 3). Interestingly, for intentional mind wandering, although a similar scaling across test difficulty levels was observed, we found no associated memory deficit when participants completed an easy forced-choice recognition test. This could mean that some forms of mind wandering (i.e., unintentional) are more likely to impair future memory performance across tests varying in difficulty or demand. In contrast, other forms of mind wandering (i.e., intentional) impair memory only for tests that rely on more detailed representations (e.g., hard forced-choice recognition and cued-recall). To explain this disparity, perhaps when individuals consciously choose to engage in mind wandering (i.e., intentional mind wandering), they may attempt to align their mind wandering with the times at which they feel subsequent memory will be the least impacted. For example, participants could choose to mind wandering after feeling they have sufficiently encoded each word pair. By this view, participants attempt to encode each item to a criterion threshold. For some items, this threshold may be reached fairly quickly, such that participants decide to engage in off-task thought for the remainder of the 5 s presentation window. Such a strategy may spare memory performance when the test can be completed on the basis of familiarity, as any additional elaborative encoding may provide a little benefit under these conditions (but see Zhang et al., 2021 for similar mnemonic costs for intentional and unintentional mind-wandering). However, such a strategy could prove costly when the test relies on more verbatim (e.g., forced-choice recognition with semantically related lures) and/or associative information (e.g., cued-recall) because further elaboration on each item may benefit memory in these contexts.

By contrast, unintentional forms of mind wandering by their nature come about without conscious intent. In these cases, it would not be possible for an individual to selectively choose an appropriate time to engage in mind wandering. As we saw in the combined analyses, this lack of control may lead to memory deficits regardless of the ease or format of an upcoming test. That being said, some evidence suggests that unintentional mind wandering may not universally affect memory performance (Thomson, Smilek, & Besner, 2014). However, there are a few important distinctions between Thomson, Smilek, & Besner, (2014) and the current research that may reconcile these divergent results. First, Thomson, Smilek, & Besner, (2014) assessed whether the overall level of unintentional mind wandering predicted participants’ overall memory performance in an item recognition test, such that the rate of mind wandering and memory performance was compared across individuals. Therefore, the finding that unintentional mind wandering only affects memory performance in some contexts (i.e., deep encoding tasks) could reflect the possibility that individuals who mind wander more may also have poorer memory in general, and that this relation is most apparent when the nominal encoding task is more attentionally demanding. The current research, however, used a within-subject comparison such that the mnemonic impact of unintentional mind wandering was measured across trials within participants. That is, for each participant, we assessed an item’s likelihood of being remembered when that participant did and did not report unintentional mind wandering. This approach factors out any trait-level differences in mind wandering propensity and general memory ability. Second, as suggested by the results of Thomson, Smilek, & Besner, (2014), certain encoding conditions (i.e., shallow encoding) may mask the cost of mind wandering on subsequent memory. Unlike Thomson, Smilek, & Besner, (2014), however, the current research did not vary the encoding task across conditions. In fact, it is likely that participants engaged in meaning-based (or deep) encoding in our experiments, given that instructions for both test formats emphasised the association between each word in the pair. As such, the consistent cost of unintentional mind wandering observed here aligns with the result of Thomson, Smilek, & Besner (2014). Finally, the memory tests employed here were likely more challenging than the item recognition tests used by Thomson, Smilek, & Besner (2014). Even for the easy forced-choice recognition test used here, participants were likely relying on their memory for the association between the cue and target, which imposes a greater demand on memory than item recognition, as the latter requires only an acontextual familiarity signal. Therefore, we acknowledge that some memory tests that require minimal levels of specificity, perhaps in combination with shallow encoding, could well be spared from the costs of mind wandering, even if done so unintentionally.

One final point to consider is the mnemonic cost of media multitasking. Unlike mind wandering, we observed a consistent memory deficit associated with media multitasking. However, such a finding is not particularly surprising given that participants would likely be devoting most, if not all, of their attentional resources towards an external device, resulting in poor encoding. That is, where while mind wandering, an individual is perceptually decoupled from the current task, while media multitasking, they may not even be looking at the task, preventing even perceptual match from contributing to later memory retrieval.

### Limitations and future directions

Although the current research has provided some important insights on mind wandering’s impact on future test performance, there exist some limitations that future research should seek to address. First, while there are numerous test formats that are used in both applied and laboratory settings, the current research only explored the impact of inattention on cued-recall and forced-choice recognition. The reasons for employing these specific test formats were twofold. First, the choice to use cued-recall served to eliminate the possibility of variation in participants’ recall criterion (Koriat & Goldsmith, 1996). And second, we chose to employ a forced-choice recognition test, rather than an item recognition test, so as to have a common scale between the two test formats utilised (i.e., proportion correct). Importantly however, although our decision to use these specific test formats is beneficial for interpreting our results, it does not allow us to draw conclusions regarding other test formats. Therefore, future research should seek to assess individuals’ capacity to adapt their mind wandering when preparing for different test formats and how such engagement in off-task thoughts may affect future memory performance.

A second limitation also relates to the properties of the specific test formats used. Specifically, because we chose to utilise a forced-choice recognition test, one of the easiest test formats, it is possible that memory performance on this test may approach ceiling level performance. Although the argument could be made that such high performance may make it difficult to observe any cost of mind wandering, this is consistent with our general point. That is, utilising a test format associated with such high performance helps to demonstrate (a) that the magnitude of the mnemonic cost of mind wandering may not be the same for all test formats and (b) that mind wandering (at least when unintentional) can hurt memory performance for even the easiest memory tests.

Finally, as alluded to previously, because test format and test difficulty are typically confounded (i.e., some test formats are inherently easier than others), we cannot say whether it is in fact the format or the difficulty of the upcoming test that determines the mnemonic cost of mind wandering. Importantly, however, even if we had equated the difficulty of the forced-choice recognition and cued-recall tests used in the current research, it is unclear whether the modifications required to equate the difficulty of these test formats would compromise the ecological validity of our results. Note that in most real-world testing scenarios, recognition will almost always be easier than cued-recall, and that artificially equating the difficulty of these formats experimentally may not accurately reflect testing conditions in any practical sense. As suggested by the current research, perhaps the most critical factor determining the cost of mind wandering is the specific demands required at the time of the test. Rather than focusing on the distinction between format and difficulty, it is likely more informative to consider the specific memory demands imposed by different tests. For example, the amount of specificity required during a cued-recall test, or any test that is generative in nature, is usually greater than that required by a forced-choice recognition test. Therefore, rather than focusing on the specific test format employed, future research may seek to explore how mind wandering affects memory on tests that differ in their dependence on details and representational specificity.

## Conclusion

Together, the findings reported here provide important new insights into the impact of off-task thought on future memory performance and constrain our understanding of the strategic regulation of inattention in the context of learning and memory. Although we found that individuals do not appear to engage in off-task thought more or less frequently depending on anticipated test demands, we did find that engagement in off-task thought negatively affects memory in most cases. Furthermore, memory performance was impacted differently depending on the amount of precision and detail required to support memory retrieval at the time of the test. These findings suggest that the mnemonic costs associated with mind wandering and other forms of inattention are not necessarily uniform but context-dependent.

## Supplemental Material

sj-docx-1-qjp-10.1177_17470218231187892 – Supplemental material for Examining the effect of expected test format and test difficulty on the frequency and mnemonic costs of mind wandering Supplemental material, sj-docx-1-qjp-10.1177_17470218231187892 for Examining the effect of expected test format and test difficulty on the frequency and mnemonic costs of mind wandering by Skylar J Laursen, Jeffrey D Wammes and Chris M Fiacconi in Quarterly Journal of Experimental Psychology

## Notes

1 Given that our sample included participants from different populations (i.e., University of Guelph students and individuals from Prolific), we first conducted all analyses including sampling population as a factor. We observed two differences between our sampling populations. First, considering rates of mind wandering, we found that University of Guelph students reported higher rates of unintentional mind wandering (*M* = .276, *SD* = .241) than individuals from Prolific (*M* = .207, *SD* = .216), *F*(1,98) = 4.002, *p* = .048, *η_p_^2^* = .040, 95% CI = [.00, .14]. Second, considering memory performance following on-task and unintentional mind-wandering probe responses, we observed a significant interaction between Test Format and Sampling Population such that the effect of Test Format was larger for University of Guelph students, *z* = 18.259, *p* < .001, Cohen’s *d* = 1.808, 95% CI = [1.612, 2.00], than for individuals from Prolific, *z* = 14.199, *p* < .001, Cohen’s *d* = 1.511, 95% CI = [1.302, 1.721]. Importantly, we observed no differences between our sampling populations regarding how expected test format affect rates of mind wandering, or how mind wandering affects future test performance. Therefore, we elected to report our analyses in text, omitting sampling population as a factor.

2 We first created our models with the maximal random-effects structure and then removed random slope parameters until our models produced a non-singular fit (see Barr et al., 2013). We made every attempt to retain the slope variable for our fixed-effect term that was of greatest interest. When this was not possible, however, we verified that for the model with and without the random slope, our variable of interest did not differ.

3 Unlike Experiment 1, in Experiment 2, participants were able to indicate when they were media multitasking. It is possible that including this response option decreased the frequency with which participants reported other forms of off-task thought compared with Experiment 1. To verify that reports of mind wandering did not change from Experiment 1 to Experiment 2, we conducted additional independent-sample *t*-test comparisons to assess any differences in the frequency of overall, intentional, and unintentional mind wandering, ignoring test format/difficulty and cycle. These analyses showed that rates of overall mind wandering were marginally greater in Experiment 1 (*M* = .324, *SD* = .225) than Experiment 2 (*M* = .270, *SD* = .181), *t*(198) = 1.869, *p* = .063, Cohen’s *d* = .270, 95% CI = [−.010, .550], rates of intentional mind wandering were significantly greater in Experiment 1 (*M* = .078, *SD* = .104) than Experiment 2 (*M* = .051, *SD* = .069), *t*(198) = 2.193, *p* = .030, Cohen’s *d* = .310, 95% CI = [.030, .590], and rates of unintentional mind wandering were not different in Experiment 1 (*M* = .246, *SD* = .173) than Experiment 2 (*M* = .220, *SD* = .157), *t*(198) = 1.136, *p* = .257, Cohen’s *d* = .160, 95% CI = [−.120, .440]. Critically, however, this is not entirely surprising given the difference in the number of study-test cycles in Experiment 1 and 2. Recall that Experiment 1 included six study-test cycles (i.e., three of each test format), whereas Experiment 2 only included two study-test cycles. In addition, because we found that rates of mind wandering increased across cycles in Experiment 1, it is possible that these differences in rates of overall and intentional mind wandering are driven by Cycles 2 and 3 in Experiment 1. Therefore, we also compared rates of overall, intentional, and unintentional mind wandering, ignoring test format, in the first two study-test cycles (i.e., the first cycle of each test format) in Experiment 1 to those reported in Experiment 2. All analyses were not significant, all *t*’s < 1.41 and all *p*’s > .160.

4 As in Experiment 2, because participants were able to indicate when they were media multitasking, it is possible that including this response option decreased the frequency with which participants reported other forms of off-task thought compared with Experiment 1. To verify that reports of mind wandering did not change from Experiment 1 to Experiment 3, we conducted additional independent-sample *t*-test comparisons to assess any differences in the frequency of overall, intentional, and unintentional mind wandering, ignoring test format/difficulty and cycle. These analyses showed that rates of overall, intentional, and unintentional mind wandering were significantly greater in Experiment 1 (*M_overall_* = .324, *SD_overall_* = .225; *M_intentional_* = .078, *SD_intentional_* = .104; *M_unintentional_* = .246, *SD_unintentional_* = .173) than Experiment 3 (*M_overall_* = .247, *SD_overall_* = .172; *M_intentional_* = .050, *SD_intentional_* = .091; *M_unintentional_* = .197, *SD_unintentional_* = .149), *t*(198) = 2.728, *p* = .007, Cohen’s *d* = .390, 95% CI = [.110, .670], *t*(198) = 2.029, *p* = .044, Cohen’s *d* = .290, 95% CI = [.010, .570], *t*(198) = 2.160, *p* = .032, Cohen’s *d* = .310, 95% CI = [.030, .590], respectively. Critically, however, similar to Experiment 2, this is not entirely surprising given the difference in the number of study-test cycles in Experiments 1 and 3. As in Experiment 2, Experiment 3 only included two study-test cycles, whereas Experiment 1 included six study-test cycles (i.e., three of each test format). In addition, because we found that rates of mind wandering increased across cycles in Experiment 1, it is possible that these differences in rates of overall and intentional mind wandering are driven by Cycles 2 and 3 in Experiment 1. Therefore, we also compared rates of overall, intentional, and unintentional mind wandering, ignoring test format, in the first two study-test cycles (i.e., the first cycle of each test format) in Experiment 1 to those reported in Experiment 3. All analyses were not significant, all *t*’s < 1.335 and all *p*’s > .183.
