# Supplementary material for: Examining the effect of expected test format and test difficulty on the frequency and mnemonic costs of mind wandering
Source: Q J Exp Psychol (Hove). 2023 Jul 21;77(5):1068–92. doi: 10.1177/17470218231187892 (PMC11032633; doi:10.1177/17470218231187892)
Supplement: sj-docx-1-qjp-10.1177_17470218231187892 – Supplemental material for Examining the effect of expected test format and test difficulty on the frequency and mnemonic costs of mind wandering [file sj-docx-1-qjp-10.1177_17470218231187892.docx]

Supplementary Material for:

Examining the Effect of Expected Test Format and Test Difficulty on the Frequency and Mnemonic Costs of Mind Wandering

Skylar J. Laursen^1^, Jeffrey D. Wammes^2, 3^, Chris M. Fiacconi^1^

^1^Department of Psychology, University of Guelph, Guelph, Ontario, Canada

^2^Department of Psychology, Queen’s University, Kingston, Ontario, Canada

^3^Centre for Neuroscience Studies, Queen’s University, Kingston, Ontario, Canada

Skylar J. Laursen

Email: [slaursen@uoguelph.ca](mailto:cfiaccon@uoguelph.ca)

Jeffrey D. Wammes

Email: [jeffrey.wammes@queensu.ca](mailto:jeffrey.wammes@queensu.ca)

Chris M. Fiacconi

Email: [cfiaccon@uoguelph.ca](mailto:cfiaccon@uoguelph.ca)

Supplementary Material A

Instruction and Follow-up Questions

**First study phase: Experiments 1, 2 and 3** (correct answers are bolded; additional questions/answers from Experiments 2 and 3 are italicized)

1. Which of the following would be considered intentionally off-task? Please select the correct answer on your keyboard.5 seconds
   1. I am currently thinking about the task that I am doing
   2. **I am purposely thinking about something other than the task**
   3. I am thinking about something other than the task, but I did not intentionally do this
   4. *I am using a device to view content unrelated to the task*
2. Which of the following would be considered on-task? Please select the correct answer on your keyboard.
   1. I am purposely thinking about something other than the task
   2. I am thinking about something other than the task, but I did not intentionally do this
   3. **I am currently thinking about the task that I am doing**
   4. *I am using a device to view content unrelated to the task*
3. Which of the following would be considered unintentionally off-task? Please select the correct answer on your keyboard.
   1. **I am thinking about something other than the task, but I did not intentionally do this**
   2. I am currently thinking about the task I am doing
   3. I am intentionally thinking about something other than the task
   4. *I am using a device to view content unrelated to the task*
4. *Which of the following would be considered media multi-tasking? Please select the correct answer on your keyboard.*
   1. ***I am using a device to view content unrelated to the task***
   2. *I am currently thinking about the task that I am doing*
   3. *I am thinking about something other than the task, but I did not intentionally do this*
   4. *I am purposely thinking about something other than the task*

**All study phases: Experiment 1**

1. Which of the following tests will you be preparing for? Please select the correct answer on your keyboard.
   1. Cued-Recall (I must recall the target word when presented with the cue word later on)
   2. Forced Choice Recognition (I must select the correct target word from 3 different options)

**Cued-recall test phase: Experiments 1 and 3** (correct answers are bolded; questions only asked in Experiment 1 are italicized; questions only asked in Experiment 3 are underlined)

1. What word from the word pair will be presented to you? Please select the correct answer on your keyboard.
   1. Both (cue and target)
   2. **Cue (left word)**
   3. Neither
   4. Target (right word)
2. Which word from the word pair will you be asked to recall? Please select the correct answer on your keyboard.
   1. Cue (left word)
   2. Neither
   3. **Target (right word)**
   4. Both (cue and target)
3. *Will you be presented with all cue words from the previous study phase? Please select the correct answer on your keyboard.*
   1. ***Yes***
   2. *No*
4. What will be presented in the top right part of the screen? Please select the correct answer on your keyboard.
   1. **The 2 first letters of the target word**
   2. The correct target word
   3. The last 2 letters of the target word

**Forced-choice recognition test phase: All Experiments** (correct answers are bolded)

1. Which word will you be asked to select? Please select the correct answer on your keyboard.
   1. The cue word that was previously presented with the target word
   2. The middle word
   3. **The target word that was previously presented with the cue word**
2. How do you select the top, middle, and bottom words? Please select the correct answer on your keyboard.
   1. Press A for the top word, B for the middle word, and C for the bottom word
   2. **Press 1 for the top word, 2 for the middle word, and 3 for the bottom word**
   3. Press 3 for the top word, 2 for the middle word, and 1 for the bottom word

**Follow-up: All Experiments**

1. During the experiment, did you write anything down to help with your memory performance (i.e., during the study phases did you write down any of the word pairs)? Please select whichever answer applies to you.
   1. Yes
   2. No
2. Did you have any interruptions during your participation today (i.e., did you get distracted by your phone, another person in the room, or leave at any time)? Please select whichever answer applies to you.
   1. No (I had no interruptions during the experiment)
   2. Yes (I was interrupted by my phone or another person in the room)
   3. Yes (I left at some point to do something else)

Supplementary Material B

Stimuli used in all Experiments

Table B1

*Stimuli used in Experiment 1*

| List 1 | List 2 | List 3 | List 4 | List 5 | List 6 |
| --- | --- | --- | --- | --- | --- |
| educator – pencil | anchor – navy | earth – sphere | pine – oak | brain – gray | lemon – sun |
| tent – forest | wound – aid | iron – engineer | vice – deputy | highway – urban | oxygen – plant |
| key – bone | customer – window | beg – knee | corn – whiskey | victim – cell | palace – servant |
| cocktail – tip | deer – shot | widow – estate | fur – nature | hospital – cure | ghost – sheet |
| freight – railroad | kingdom – tyranny | pressure – vein | quiet – ear | evidence – tape | author – series |
| debate – ballot | milk – jersey | moon – phase | cow – butter | grass – blade | swimming – tide |
| clover – rose | prairie – rural | dozen – bread | valley – pit | produce – orange | youth – calf |
| industry – fuel | physics – mass | steak – ham | wool – shear | division – sum | sky – ocean |
| heaven – wing | office – suit | oil – bird | weather – mercury | liver – stomach | fork – bowl |
| garden – bloom | gulf – sea | captain – cockpit | blonde – brush | cake – pie | chlorine – fish |
| coal – gas | eye – contact | coach – goal | leaf – bush | length – weight | arm – foot |
| horse – chicken | taste – hear | judge – prison | dairy – utter | palm – sole | reward – treat |
| button – pocket | growth – increase | carbon – atom | sodium – tin | nation – minister | golf – driver |
| prime – five | alter – marriage | potato – liquor | idle – hazard | leather – skin | spade – club |
| stroke – heart | scream – horror | infant – carriage | cancer – tobacco | dollar – cent | knight – crown |
| mineral – salt | march – army | basement – ground | dawn – midnight | east – needle | tunnel – bridge |
| breast – food | paper – bark | mine – drill | organ – doctor | stop – veto | stable – farm |
| pollen – honey | picture - gallery | mortal – death | fence – border | fruit – bunch | composer – violin |
| quest – vacation | soap – vacuum | smell – vision | wrong - red | parade – ceremony | tree – ring |
| surprise – balloon | bed – river | teacher – board | lens – sight | dentist – filling | antique – century |
| map – capital | copper – green | pen – mark | branch – money | alien – star | alcohol – clean |
| wax – beard | bite – lake | accident – broken | fabric – fashion | dinner – cereal | moisture – health |
| coffee – morning | drug – angel | drama – costume | naval – belly | dream – fatigue | air – fog |
| hot – shower | gold – fool | muzzle – harness | drain – snake | platform – purse | couch – channel |
| bank – flow | bat – echo | beach – burn | bee – jacket | coast – edge | flock – egg |
| square – addition | candy – holiday | appeal – witness | match – candle | congress – debt | college – keen |
| election – citizen | letter – grade | writing – library | gift – free | blood – faint | fair – charming |
| fire – flood | fort – king | penny – fountain | flower – period | game – zone | yard – football |
| straw – blue | knife – stone | lime – beer | science – reaction | party – vote | mustard – pepper |
| quarter – silver | question – learn | beef – rare | brother – uncle | ripe – nose | sunlight – rotation |
| height – scale | shell – shore | aunt – sister | finish – track | stern – port | envelope – mail |
| jack – tire | movie – scene | cottage – summer | fault – plate | hero – save | crime – lawyer |
| cut – iodine | market – merchant | travel – mileage | musician – painter | medicine – opium | mother – patient |
| plot – theme | amateur – recruit | shop – register | stadium – receiver | sugar – bitter | toast – wine |
| guide – tourist | tragedy – comedy | radio – tune | bubble – clear | flame – ash | blanket – nursery |
| circuit – race | incident – claim | smoke – crawl | bubble – clear | flush – straight | collar – jail |
| degree – scholar | ditch – curb | dog – friend | duke – earl | entrance – debut | fly – camp |
| marble – ivory | lunch – machine | loan – mortgage | camera – moment | news – evening | pasture – herd |
| mare – pony | protest – passion | peace – troop | paint – polish | battle – quarrel | bishop – queen |
| road – permit | root – floor | audience – speech | wheel – spoke | suspect – plea | student – test |
| vacant – suite | velocity – volume | vertical – slope | worker – union | teach – academy | animal – meat |
| black – darkness | budget – account | older – cane | chair – boss | calm – storm | orderly – assist |
| cash – tax | closet – cellar | evaluate – fail | zero – empty | grave – grief | military – fleet |
| harvest – autumn | child – park | kiss – tongue | sergeant – leader | lumber – cabin | win – majority |
| minute – clock | muscle – nerve | saddle – ride | daylight – shadow | tea – steam | hardware – build |

Table B2

*Results of comparisons made for LSA values between lists in Experiment 1, descriptive statistics included*

|  | List 1 |  | List 2 |  | List 3 |  | List 4 |  | List 5 |  | List 6 |
| --- | --- | --- | --- | --- | --- | --- | --- | --- | --- | --- | --- |
|  | *M* = 0.14  *SD* = 0.20 |  | *M* = 0.14  *SD* = 0.17 |  | *M* = 0.16  *SD* = 0.21 |  | *M* = 0.13  *SD* = 0.18 |  | *M* = 0.13  *SD* = 0.15 |  | *M* = 0.14  *SD* = 0.17 |
| Effect of List | *F*(5, 264) = 0.14, *p* = .98 | | | | | | | | | | |

*Note. M* and *SD* represent mean and standard deviation, respectively.

Table B3

*Stimuli used in Experiments 2 and 3*

| List 1 | | |  | List 2 | | |
| --- | --- | --- | --- | --- | --- | --- |
| Cue – Target | Lure 1 | Lure 2 |  | Cue – Target | Lure 1 | Lure 2 |
| educator – pencil | teacher | grade |  | kingdom – tyranny | empire | citizen |
| tent – forest | camp | park |  | milk – jersey | cake | dairy |
| key – bone | lock | bronze |  | prairie – rural | village | farm |
| cocktail – tip | bar | drink |  | physics – mass | measure | graph |
| freight – railroad | train | rail |  | office – suit | tie | boss |
| debate – ballot | election | vote |  | gulf – sea | lake | pond |
| heaven – wing | angel | hell |  | taste – hear | smell | sight |
| garden – bloom | meadow | spring |  | growth – increase | gain | combine |
| button – pocket | belt | jacket |  | alter – marriage | ring | wedding |
| prime – five | nine | eight |  | scream – horror | fear | terror |
| breast – food | baby | mother |  | march – army | command | soldier |
| pollen – honey | bee | insect |  | picture - gallery | portrait | camera |
| quest – vacation | paradise | holiday |  | soap – vacuum | sweep | clean |
| surprise – balloon | party | gift |  | bed – river | rush | rapid |
| map – capital | south | coast |  | copper – green | brass | gold |
| wax – beard | candle | body |  | question – learn | answer | learning |
| coffee – morning | café | tea |  | shell – shore | swim | shallow |
| hot – shower | steam | sweat |  | movie – scene | film | show |
| fire – flood | hail | disaster |  | tragedy – comedy | drama | romance |
| quarter – silver | dollar | coin |  | incident – claim | lawyer | sue |
| height – scale | size | heavy |  | ditch – curb | lane | route |
| jack – tire | car | spare |  | lunch – machine | market | meal |
| plot – theme | play | act |  | protest – passion | proud | peace |
| circuit – race | course | path |  | velocity – volume | vertical | vessel |
| degree – scholar | graduate | academy |  | budget – account | finance | loan |
| marble – ivory | silk | crystal |  | widow – estate | husband | loss |
| mare – pony | horse | stable |  | pressure – vein | nerve | muscle |
| road – permit | vehicle | permit |  | moon – phase | flag | land |
| vacant – suite | hotel | guest |  | dozen – bread | dough | half |
| black – darkness | midnight | dark |  | steak – ham | beef | pork |
| minute – clock | hour | watch |  | oil – bird | bat | bear |
| anchor – navy | ship | fleet |  | captain – cockpit | plane | control |
| wound – aid | scar | injury |  | coach – goal | team | opponent |
| customer – window | register | store |  | muzzle – harness | collar | walk |
| deer – shot | shoot | aim |  | beach – burn | sand | bay |
| earth – sphere | planet | globe |  | appeal – witness | justice | court |
| iron – engineer | metal | builder |  | radio – tune | channel | speaker |
| beg – knee | poor | pray |  | audience – speech | spark | crowd |
| judge – prison | plea | jail |  | kiss – tongue | cheek | lip |
| basement – ground | cellar | floor |  | fur – nature | animal | creature |
| drill – mine | mineral | stone |  | quiet – ear | whisper | silence |
| accident – broken | break | broke |  | cow – butter | utter | barn |
| writing – library | essay | reading |  | valley – pit | mountain | deep |
| penny – fountain | cent | fortune |  | wool – shear | sheep | cotton |
| aunt – sister | cousin | wife |  | leaf – bush | root | stem |
| travel – mileage | mile | trip |  | fence – border | boundary | edge |
| smoke – crawl | cancer | cigar |  | wrong - red | error | fail |
| older – cane | calm | age |  | drain – snake | plug | sink |
| saddle – ride | rode | jump |  | brother – uncle | father | boy |
| pine – oak | tree | cone |  | fault – plate | mantle | core |
| vice – deputy | chief | officer |  | musician – painter | artist | singer |
| corn – whiskey | alcohol | wine |  | dear – message | letter | wrote |
| weather – mercury | forecast | report |  | paint – polish | paste | sketch |
| blonde – brush | hair | beauty |  | wheel – spoke | round | turn |
| sodium – tin | aluminum | carbon |  | zero – empty | removal | decay |
| lens – vision | pupil | contact |  | sergeant – leader | lead | troop |
| fabric – fashion | clothing | style |  | daylight – shadow | dawn | sunlight |
| science – reaction | research | solution |  | grass – blade | lawn | trim |
| bubble – clear | foam | wash |  | produce – orange | fruit | juice |
| duke – earl | lord | priest |  | division – sum | addition | result |
| worker – union | uniform | unite |  | length – weight | pound | inch |
| brain – gray | genius | smart |  | palm – sole | shoe | shoulder |
| highway – urban | fast | tourist |  | leather – skin | flesh | meat |
| victim – cell | suspect | law |  | stop – veto | halt | pause |
| hospital – cure | doctor | care |  | dentist – filling | teeth | jaw |
| evidence – tape | crime | trial |  | dream – fatigue | rest | sleep |
| liver – stomach | heart | organ |  | stern – port | bow | boat |
| nation – minister | cabinet | chair |  | medicine – opium | drug | needle |
| parade – ceremony | occasion | toast |  | sugar – bitter | sweet | candy |
| alien – star | space | stranger |  | flame – ash | spark | coal |
| platform – purse | skirt | pump |  | entrance – debut | welcome | arrival |
| congress – debt | council | mayor |  | news – evening | story | paper |
| blood – faint | stroke | panic |  | lumber – cabin | lodge | cottage |
| hero – save | rescue | safe |  | ghost – sheet | pale | costume |
| battle – quarrel | enemy | fight |  | author – series | book | novel |
| grave – grief | death | burial |  | swimming – tide | pool | wave |
| lemon – sun | lime | summer |  | youth – calf | child | junior |
| oxygen – plant | breath | air |  | sky – ocean | blue | cloud |
| palace – servant | prince | staff |  | fork – bowl | knife | cup |
| arm – foot | leg | elbow |  | reward – treat | trick | ribbon |
| golf – driver | hole | club |  | fair – charming | princess | elegant |
| tunnel – bridge | gate | arch |  | mustard – pepper | onion | salt |
| composer – violin | piano | music |  | envelope – mail | package | deliver |
| antique – century | million | history |  | bishop – queen | knight | king |
| flock – egg | hen | chicken |  | student – test | subject | teach |
| college – keen | semester | lecture |  | orderly – assist | nurse | patient |
| yard – football | stadium | receiver |  | win – majority | champion | victory |
| blanket – nursery | bottle | infant |  | clover – rose | flower | grove |
| pasture – herd | cattle | ranch |  | industry – fuel | factory | gas |
| hardware – build | building | brick |  | cash – tax | money | payment |

Table B4

*Results of comparisons made for LSA values between lists in Experiments 2 and 3, descriptive statistics included*

|  | List 1 |  | List 2 |
| --- | --- | --- | --- |
|  | *M* = 0.16 |  | *M* = 0.13 |
|  | *SD* = 0.20 |  | *SD* = 0.16 |
| Effect of  List | *t*(358) = 0.18, *p* = .85 | | |

*Note. M* and *SD* represent mean and standard deviation, respectively.

Supplementary Material C

Mind Wandering Description

At multiple times throughout the study phase we will ask you a question about your mental state. Specifically, we will ask you if you are on-task, intentionally off-task, unintentionally off-task, or media multi-tasking.

It's perfectly normal to think about things that are not related to the task. For instance, you may think about off-task things such as something you did recently or will be doing later, your current emotional or physical state, your personal worries, daydreams, or your external environment.

**As well, it is also common for individuals to use external devices (e.g., smart phone, tablet, computer) to view context that is unrelated to the task. This could include browsing social media, responding to texts, or watching unrelated videos.

We ask that you try to refrain from using these external devices as much as possible, but it is okay if this does happen. Please try your best to honestly assess your thoughts and choose a response that best describes your thoughts at the time when we ask.

The four response options include:

ON TASK: This occurs when your full attention is on the task you are currently completing.

INTENTIONAL: Sometimes when you're working on a task, you might deliberately or intentionally think about things unrelated to the task. This is what we refer to as intentional off-task thinking. That is, when you deliberately think about something other than the task.

UNINTENTIONAL: Other times when you're working on a task, you might unintentionally think about things unrelated to the task. This is what we refer to as unintentional off-task thinking. That is, when you spontaneously think about something other than the task.

**MEDIA MULTI-TASKING: Other times when you’re working on a task, you might find yourself using your devices, such as your computer, smartphone, or tablet, to view content unrelated to the task. This is what we refer to as media multi-tasking. That is, when you are using a device to view content unrelated to the task.

**These descriptions were only provided in Experiments 2 and 3

Supplementary Material D

Mixed-effects Models and Relevant Test Statistics

**Experiment 1**

**Model D1.** Used to compare the relation between memory performance for on-task and mind wandering probe responses for cued-recall and forced-choice recognition tests in Experiment 1 (see Table D1 for relevant test statistics)

glmer(Memory ~ Probe Response * Test Format + (1 + Probe Response + Test Format | Subject) + (1 | Word Pair), control = glmerControl(optimizer = “bobyqa”), family = binomial)

**Model D2.** Used to compare the relation between memory performance for on-task, intentionally off-task, and unintentionally off-task probe responses for cued-recall and forced-choice recognition tests in Experiment 1 (see Table D1 for relevant test statistics)

glmer(Memory ~ Probe Response * Test Format + (1 | Subject) + (1 | Word Pair), control = glmerControl(optimizer = “bobyqa”), family = binomial)

Table D1.

*All relevant test statistics for the evaluation of Models D1 and D2*

|  | Overall Mind Wandering | | |
| --- | --- | --- | --- |
|  | *OR* [95% CI] | *χ^2^* | *p* |
| Probe Response | 0.595 [0.519, 0.667] | 61.892 | < .001 |
| Test Format | 4.610 [ 4.054, 5.451] | 419.645 | < .001 |
| Interaction between  Probe Response and  Test Format | 1.065 [0.941, 1.220] | 1.040 | .308 |
|  | Intentional Mind Wandering | | |
|  | *OR* [95% CI] | *χ^2^* | *p* |
| Probe Response | 0.589 [0.483, 0.740] | 23.734 | < .001 |
| Test Format | 5.729 [4.651, 7.130] | 236.042 | < .001 |
| Interaction between  Probe Response and  Test Format | 1.310 [1.094, 1.640] | 6.878 | .009 |
|  | Unintentional Mind Wandering | | |
|  | *OR* [95% CI] | *χ^2^* | *p* |
| Probe Response | 0.626 [0.540, 0.715] | 55.003 | < .001 |
| Test Format | 4.557 [3.941, 5.296] | 500.504 | < .001 |
| Interaction between  Probe Response and  Test Format | 1.089 [0.968, 1.238] | 2.064 | .151 |

Note. *OR* values represent odds ratios that have been converted from log(odds). 95% CI were calculated using bootstrapping method

**Experiment 2**

**Model D3.** Used to compare the relation between memory performance for on-task, off-task, unintentionally off-task, and media multi-tasking probe responses for easy and difficult forced-choice recognition tests in Experiment 2 (see Table D2 for relevant test statistics)

glmer(Memory ~ Probe Response * Test Difficulty + (1 | Subject) + (1 | Word Pair), control = glmerControl(optimizer = “bobyqa”), family = binomial)

**Model D4.** Used to compare the relation between memory performance for on-task and intentionally off-task probe responses for easy and difficult forced-choice recognition tests in Experiment 2 (see Table D2 for relevant test statistics)

glmer(Memory ~ Probe Response * Test Difficulty + (1 + Probe Response + Test Difficulty | Subject) + (1 | Word Pair), control = glmerControl(optimizer = “bobyqa”), family = binomial)

Table D2.

*All relevant test statistics for the evaluation of Models D3 and D4*

|  | Overall Mind Wandering | | |
| --- | --- | --- | --- |
|  | *OR* [95% CI] | *χ^2^* | *p* |
| Probe Response | 0.791 [0.688,0.901] | 14.156 | < .001 |
| Test Difficulty | 1.748 [1.539,1.965] | 90.756 | < .001 |
| Interaction between  Probe Response and  Test Difficulty | 1.019 [0.901, 1.149] | 0.104 | .747 |
|  | Intentional Mind Wandering | | |
|  | *OR* [95% CI] | *χ^2^* | *p* |
| Probe Response | 0.854 [0.642, 1.339] | 0.767 | .381 |
| Test Difficulty | 2.063 [1.577, 3.196] | 18.968 | < .001 |
| Interaction between  Probe Response and  Test Difficulty | 1.183 [0.915, 1.823] | 1.034 | .309 |
|  | Unintentional Mind Wandering | | |
|  | *OR* [95% CI] | *χ^2^* | *p* |
| Probe Response | 0.786 [0.697, 0.897] | 13.484 | < .001 |
| Test Difficulty | 1.714 [1.531, 1.949] | 75.265 | < .001 |
| Interaction between  Probe Response and  Test Difficulty | 1.001 [0.863, 1.147] | .0002 | .989 |
|  | Media Multi-Tasking | | |
|  | *OR* [95% CI] | *χ^2^* | *p* |
| Probe Response | 0.684 [0.517, 0.917] | 8.422 | .004 |
| Test Difficulty | 1.875 [1.470, 2.483] | 26.351 | < .001 |
| Interaction between  Probe Response and  Test Difficulty | 1.089 [0.864, 1.414] | 0.491 | .483 |

Note. *OR* values represent odds ratios that have been converted from log(odds). 95% CI were calculated using bootstrapping method

**Experiment 3**

**Model D5.** Used to compare the relation between memory performance for on-task, off-task, intentionally off-task, and unintentionally off-task probe responses for difficult forced-choice recognition and easy cued-recall tests in Experiment 3 (see Table D3 for relevant test statistics)

glmer(Memory ~ Probe Response * Test Format + (1 + Probe Response + Test Format | Subject) + (1 | Word Pair), control = glmerControl(optimizer = “bobyqa”), family = binomial)

**Model D6.** Used to compare the relation between memory performance and probe response (i.e., on-task, media multi-tasking) for difficult forced-choice recognition and easy cued-recall tests in Experiment 3 (see Table D3 for relevant test statistics)

glmer(Memory ~ Probe Response * Test Format + (1 + Probe Response | Subject) + (1 | Word Pair), control = glmerControl(optimizer = “bobyqa”), family = binomial)

Table D3.

*All relevant test statistics for the evaluation of Models D5 and D6*

|  | Overall Mind Wandering | | |
| --- | --- | --- | --- |
|  | *OR* [95% CI] | *χ^2^* | *p* |
| Probe Response | 0.709 [0.612, 0.822] | 21.148 | < .001 |
| Test Format | 1.977 [1.715, 2.241] | 89.892 | < .001 |
| Interaction between  Probe Response and  Test Format | 1.038 [0.915, 1.167] | 0.346 | .557 |
|  | Intentional Mind Wandering | | |
|  | *OR* [95% CI] | *χ^2^* | *p* |
| Probe Response | 0.620 [0.419, 0.890] | 6.918 | .009 |
| Test Format | 1.992 [1.482, 2.718] | 20.597 | < .001 |
| Interaction between  Probe Response and  Test Format | 1.044 [0.794, 1.425] | 0.085 | .771 |
|  | Unintentional Mind Wandering | | |
|  | *OR* [95% CI] | *χ^2^* | *p* |
| Probe Response | 0.712 [0.620, 0.819] | 24.870 | < .001 |
| Test Format | 1.935 [1.609, 2.251] | 80.104 | < .001 |
| Interaction between  Probe Response and  Test Format | 1.020 [0.882, 1.176] | .088 | .767 |
|  | Media Multi-Tasking | | |
|  | *OR* [95% CI] | *χ^2^* | *p* |
| Probe Response | 0.617 [0.436, 0.853] | 9.648 | .002 |
| Test Format | 1.772 [1.351, 2.573] | 14.880 | < .001 |
| Interaction between  Probe Response and  Test Format | 0.955 [0.729, 1.393] | 0.095 | .757 |

Note. *OR* values represent odds ratios that have been converted from log(odds). 95% CI were calculated using bootstrapping method

**Combined Analyses**

**Model D7.** Used to compare the relation between memory performance for on-task, off-task, and intentionally off-task probe responses in the Combined analysis (see Table D4 for relevant test statistics)

glmer(Memory ~ Probe Response * Test Format + (1 + Probe Response | Subject) + (1 | Word Pair), control = glmerControl(optimizer = “bobyqa”), family = binomial)

**Model D8.** Used to compare the relation between memory performance for on-task, unintentionally off-task and media multi-tasking probe responses in the Combined analysis (see Table D4 for relevant test statistics)

glmer(Memory ~ Probe Response * Test Format + (1 | Subject) + (1 | Word Pair), control = glmerControl(optimizer = “bobyqa”), family = binomial)

Table D4

*All relevant test statistics for the evaluation of the simple effect of Probe Response for Models D7 and D8.*

|  | Overall Mind Wandering | | |
| --- | --- | --- | --- |
|  | *OR* [95% CI] | *z* | *p* |
| Easy Forced-choice Recognition | 0.539 [0.414, 0.702] | -4.591 | < .001 |
| Difficult Forced-choice Recognition | 0.552 [0.435, 0.699] | -4.929 | < .001 |
| Easy Cued-recall | 0.509 [0.362, 0.715] | -3.898 | < .001 |
| Difficult cued-recall | 0.323 [0.241, 0.434] | -7.511 | < .001 |
|  | Intentional Mind Wandering | | |
|  | *OR* [95% CI] | *z* | *p* |
| Easy Forced-choice Recognition | 0.728 [0.410, 1.295] | -1.081 | .280 |
| Difficult Forced-choice Recognition | 0.502 [0.303, 0.831] | -2.681 | .007 |
| Easy Cued-recall | 0.421 [0.189, 0.940] | -2.110 | .035 |
| Difficult cued-recall | 0.197 [0.104, 0.373] | -4.983 | < .001 |
|  | Unintentional Mind Wandering | | |
|  | *OR* [95% CI] | *z* | *p* |
| Easy Forced-choice Recognition | 0.548 [0.424, 0.708] | 4.589 | < .001 |
| Difficult Forced-choice Recognition | 0.579 [0.455, 0.737] | 4.429 | < .001 |
| Easy Cued-recall | 0.498 [0.349, 0.709] | 3.866 | < .001 |
| Difficult cued-recall | 0.340 [0.252, 0.459] | 7.041 | < .001 |
|  | Media Multi-Tasking | | |
|  | *OR* [95% CI] | *z* | *p* |
| Easy Forced-choice Recognition | 0.590 [0.296, 1.179] | -1.494 | .135 |
| Difficult Forced-choice Recognition | 0.393 [0.229, 0.672] | -3.413 | < .001 |
| Easy Cued-recall | 0.454 [0.202, 1.023] | -1.905 | .057 |

Note. *OR* values represent odds ratios that have been converted from log(odds) and reflect the reduced odds of accurate memory when reportedly off-task relative to on-task. 95% CI were calculated using bootstrapping method
